# Biphasic cell cycle defect causes impaired neurogenesis in down syndrome

**DOI:** 10.3389/fgene.2022.1007519

**Published:** 2022-10-12

**Authors:** Vishi Sharma, Sunita Nehra, Long H. Do, Anwesha Ghosh, Aniruddha J. Deshpande, Nishant Singhal

**Affiliations:** ^1^ National Centre for Cell Science, Pune, India; ^2^ Department of Neuroscience, University of California, San Diego, San Diego, CA, United States; ^3^ Sanford Burnham Prebys Medical Discovery Institute, La Jolla, CA, United States

**Keywords:** down syndrome, neurogenesis, cell cycle, disease modeling, mouse iPSCs, human iPSCs, Pax6 (RRID: AB_1566562; RRID: AB_2565003; RRID: AB_291612), trisomy 21

## Abstract

Impaired neurogenesis in Down syndrome (DS) is characterized by reduced neurons, increased glial cells, and delayed cortical lamination. However, the underlying cause for impaired neurogenesis in DS is not clear. Using both human and mouse iPSCs, we demonstrate that DS impaired neurogenesis is due to biphasic cell cycle dysregulation during the generation of neural progenitors from iPSCs named the “neurogenic stage” of neurogenesis. Upon neural induction, DS cells showed reduced proliferation during the early phase followed by increased proliferation in the late phase of the neurogenic stage compared to control cells. While reduced proliferation in the early phase causes reduced neural progenitor pool, increased proliferation in the late phase leads to delayed post mitotic neuron generation in DS. RNAseq analysis of late-phase DS progenitor cells revealed upregulation of S phase-promoting regulators, Notch, Wnt, Interferon pathways, and REST, and downregulation of several genes of the BAF chromatin remodeling complex. NFIB and POU3F4, neurogenic genes activated by the interaction of PAX6 and the BAF complex, were downregulated in DS cells. ChIPseq analysis of late-phase neural progenitors revealed aberrant PAX6 binding with reduced promoter occupancy in DS cells. Together, these data indicate that impaired neurogenesis in DS is due to biphasic cell cycle dysregulation during the neurogenic stage of neurogenesis.

## Introduction

Down syndrome (DS), trisomy 21, is a common cause of intellectual disability (ID) (Chapman and Hesketh, 2000). ID in DS is due to dysfunction at various stages of neurodevelopment. Among them, reduced neurogenesis, dendritic hypotrophy and connectivity, imbalance of excitatory glutamatergic, and inhibitory GABAergic system play a significant role (Haydar and Reeves, 2012).

At the microscopic level, the DS cortex shows fewer neurons, decreased neuronal densities, and abnormal neuronal distribution, especially in cortical layers II and IV (Wisniewski et al., 1984; Wisniewski, 1990). Examination of the visual cortex found a significantly reduced number of neurons at the time of birth, followed by recovery in the number of neurons in the DS brain compared to the control brain (Wisniewski et al., 1984). Further analysis of a fetal DS brain found ∼20–50% fewer neurons in the entorhinal cortex, the dentate gyrus of the hippocampus, hippocampal pyramidal layers, lateral parahippocampal gyrus, and presubiculum compared to the control brain (Guidi et al., 2008). In contrast to the reduced number of neurons, an increased number of astrocytes in the fetal DS brain entorhinal cortex, hippocampus, parahippocampal gyrus, and presubiculum were observed (Guidi et al., 2008; Zdaniuk et al., 2011). Additionally, the emergence of lamination is delayed and disorganized in DS (Golden and Hyman, 1994).

Current understanding of fetal DS neurogenesis is obtained mainly from fetal DS brain section studies. While these studies have significantly improved our understanding of ID in DS, these studies suffer from the limited sample size, single snapshot information, and absence of mechanistic studies. To overcome these limitations, several mouse models of DS have been generated to understand ID in DS. A recent comparative analysis of cytogenetically distinct DS mouse models Ts1Cje, Ts65Dn, and Dp (16) 1/Yey over the lifespan found that Ts65Dn mice were most consistently affected concerning somatic growth, neurogenesis, and brain morphogenesis. Of particular interest, while Ts65Dn mouse models showed reduced neocortical neurogenesis at E15.5 compared to its control littermates, Ts1Cje, and Dp (16) 1/Yey showed no changes (Aziz et al., 2018). Ts65Dn is also the most studied mouse model of DS (Reeves et al., 1995; Das and Reeves, 2011; Liu et al., 2011; Herault et al., 2017). Ts65Dn mice are segmentally trisomic for a portion of mouse chromosome 16; the segment is syntenic to a portion of the long arm of HSA21 and contains an estimated 90 mouse genes with human orthologues out of 164 non-keratin coding proteins (Davisson et al., 1993; Gupta et al., 2016). Ts65Dn mice display many DS-relevant cognitive phenotypes, including deficits in learning and behavior (Reeves et al., 1995; Faizi et al., 2011).

Similar to DS, changes in the Ts65Dn brain are present during embryonic life and beyond. The Ts65Dn adult brain shows a reduced number of neurons and increased glial cells (Contestabile et al., 2007; Contestabile et al., 2009). The Ts65Dn brain displays reduced thickness in the neocortical intermediate zone (IZ), subplate (SP), and cortical plate (CP) from E13.5 onwards. While the thickness of neocortical IZ, SP, and CP recovered by E18.5, these same zones displayed a ∼25% reduced cell density. Ts65Dn mice also showed a reduced number of neurons in layers IV, V, and VI suggesting a selective reduction in cortical neuron production. This study also found that Ts65Dn mice delayed prenatal cortex due to lengthening of S-phase and total cell cycle (Tc) in the initial phase that is at E13.5, while in the later phase of the neurogenic period that is from E14.5 onwards, the difference in Tc between euploid mice and Ts65Dn mice becomes smaller. In addition, this study found an increased number of neural progenitor cells at E16.5 in Ts65Dn SVZ compared to euploid mice (Chakrabarti et al., 2007). However, it is unclear if the increased number of neural progenitors at the late neurogenic phase observed in Ts65Dn mouse is also present during fetal DS neurogenesis, which may explain the increased number of glial cells and delayed cortical lamination as well as recovery of the number of neurons postnatally.

In addition to the development of newer mouse models (Kazuki et al., 2020), human-induced pluripotent stem cells (hiPSCs) (Takahashi and Yamanaka, 2006) (Takahashi et al., 2007) generated by reprogramming of the DS patient somatic cells have been used to explore DS impaired neurogenesis. Although earlier studies using human iPSCs have benefited us in understanding DS neurological disorder, they have generated conflicting results for impaired neurogenesis in DS, perhaps due to a lack of standardized *in vitro* neural differentiation protocols (Shi et al., 2012; Briggs et al., 2013; Lu et al., 2013; Weick et al., 2013; Hibaoui et al., 2014; Sobol et al., 2019) or variations in hiPSC lines (Rouhani et al., 2022). For instance, initial studies reported synaptic deficit in DS neurons (Weick et al., 2013) and detected amyloid plaques (Shi et al., 2012) but found normal neurogenesis in DS cells compared to control cells. Another study reported normal neural differentiation of DS cells, but at a later time point, DS cells generated more astroglia (Briggs et al., 2013). In contrast, two studies found reduced neurogenesis in DS cells (Jiang et al., 2013) (Hibaoui et al., 2014). Both of these studies found a reduced proliferation of DS cells. Jiang et al. showed that the silencing of extra chromosome 21 by XIST reversed proliferation deficit and neural rosette formation (Jiang et al., 2013). Hibaoui et al. found that treatment with EGCG, an inhibitor of DYRK1A kinase activity or shRNA against DYRK1A before starting neural induction of hiPSCs rescued DS neural differentiation (Hibaoui et al., 2014). However, these observations do not fully explain several observations like increased glial cell production, delayed cortical lamination, and recovery of the number of neurons in the visual cortex in individuals with DS postnatally. Interestingly, Hibaoui et al. also found that EGCG treatment failed to rescue DS neurogenesis when applied during neuronal differentiation. This observation indicated that molecular mechanisms causing DS impaired neurogenesis might differ in the late phase compared to the early phase of neurogenesis.

These observations from hiPSCs-based studies in combination with studies from Ts65Dn mice showing a reduced number of neural progenitor cells followed by the increased number of neural progenitor cells in the late phase intrigued us to further investigate DS neurogenesis by utilizing both Ts65Dn mouse iPSCs and DS human iPSCs. We reasoned that Ts65Dn miPSCs-based studies would allow us to compare *in vitro* differentiation data with in-depth *in vivo* studies carried out in this mouse model. Additionally, results obtained from the miPSCs-based study will guide the generation of a robust hiPSCs-based DS neurogenesis model, which will allow the identification of additional mechanisms of impaired neurogenesis in DS.

Accordingly, we carried out investigations in mouse and human iPSC cells. We found that Ts65Dn mouse iPSCs and DS human iPSCs demonstrated reduced neuronal differentiation compared to control iPSCs, consistent with *in vivo* observations. Strikingly we found that during the early phase of neural differentiation of iPSCs, DS cells undergo reduced proliferation, but in the late phase, DS neural progenitor cells show increased proliferation compared to control cells. While the reduced proliferation of neural progenitor cells in an early phase will cause a reduction in the number of DS neural progenitors, the increased proliferation causes delayed cell cycle exit leading to impaired generation of post mitotic neurons. Further, global transcriptomic analysis of late-phase human DS cells revealed widespread differences concerning isogenic euploid cells with increased expression of genes that encourage entry into and maintenance of cells in the S-phase, upregulation of the Notch, Wnt, and Interferon pathways, and upregulation of *REST.* In contrast, there was downregulation of the expression of genes whose products are involved in chromatin remodeling, including components of the BAF complex. Consistently, there was downregulation of the neurogenic genes *NFIB* and *POU3F4*, suggesting decreased activation by the PAX6 and BAF complex and marked differences in PAX6 genomic binding with reduced promoter occupancy.

In summary, our studies point to biphasic dysregulation of the cell cycle in both human and mouse model cells during neurogenesis, which may account for reduced neurons, increased glial cells, and delayed cortical lamination during DS brain development. The human DS platform established in this study will enable future studies to discover phase-specific mechanisms of defective neurogenesis in DS.

## Materials and methods

### Induced pluripotent cells derivation, culture, and characterization

Mouse embryonic fibroblasts (MEFs) were derived from E18.5 2N and Ts65Dn embryos and maintained in low glucose DMEM containing 10% FBS, 1X penicillin, streptomycin, and glutamine (PSG), 1X non-essential amino acid (NEAA) and 1X β-mercaptoethanol (βME). The experiments were approved by the University of California, San Diego, institutional animal care and use committee. The generation of mouse iPSCs and their characterization were described previously (Singhal et al., 2010). Briefly, Oct4, Sox2, Klf4, and cMyc retroviruses were used in a ratio of 3:1:1:1 to generate mouse iPSCs. Mouse iPSCs and ESD3 [D3] (ATCC® CRL 1934™) were maintained in knockout DMEM containing 15% ES qualified FBS, 1X PSG, 1X NEAA, 1X β-ME and 2000 units/ml of leukemia inhibitory factor (LIF) (Millipore) on feeder layer of mitomycin-C treated MEFs. Mouse iPSCs were characterized for pluripotency by ICC using pluripotency-associated markers Dppa2, Sox2, Oct4, and Ssea1, as described previously (Singhal et al., 2010). For *in vitro* three germ layer differentiation, mouse iPSCs were harvested by trypsinization and transferred to bacterial culture dishes in ESC medium without leukemia inhibitory factor. After 3 days in culture, aggregated cells were plated onto gelatin-coated tissue culture dishes and incubated for 5 days in the presence of 5 μM retinoic acid. Cells were stained with an anti-TUBB3 monoclonal antibody, anti-SMA antibody, or anti-AFP antibody and counterstained with DAPI (Vector). Human iPSCs were a gift from Stuart Orkin’s lab and maintained as described previously (Maclean et al., 2012; Singhal et al., 2016). Briefly, DS-hiPSCs and isogenic euploid hiPSCs were maintained on mitomycin C treated CF1 fibroblast using knockout D-MEM/F-12 containing 20% knockout serum replacement, 1X PSG, 1X NEAA, 1X βME (all Invitrogen) and 25 ng/ml human basic FGF (Peprotech #100-18B). Differentiated colonies were removed by manual curating before each passage. The expression of pluripotency markers was carried out as described previously using OCT4 and SOX2 for ICC, QRT-PCR for OCT4, SOX2, and NANOG, and *in vitro* three germ layer differentiation potential of hiPSCs using TUBB3 (ectodermal marker), SMA (mesodermal marker) and, AFP (endodermal marker) (Nehra et al., 2022; Sharma et al., 2022).

### Karyotyping

Karyotyping was carried out as described previously (Nehra et al., 2022). Briefly, hiPSCs were blocked at metaphase by exposure to 0.1 μg/ml Colcemid solution for 3 hrs. (Gibco). Karyotyping of isogenic euploid and Down syndrome hiPSCs was performed using G-banding with a resolution of 350–400, using Olympus BX51 microscope and Cytovision software (Leica).

### Neuronal differentiation

For neural differentiation of miPSCs using embryoid bodies (EBs), miPSCs were dissociated into single cells and plated on a low attachment cell culture plate in the miPSCs cell culture medium in the absence of LIF. After 4 days, EBs were cultured in suspension in the presence of 5 μM Retinoic acid (Sigma) for an additional 4 days. EBs were then plated onto poly D-lysine-laminin coated plates for three more weeks in N2B27 medium. Monolayer differentiation of mouse iPSCs was carried out as described previously (Gaspard et al., 2009). Briefly, mouse iPSCs were plated at low cell density and treated with 1 μM Cyclopamine from day 2–10 in 1x N2 and 1x B27 medium. After 12 days, the cells were re-plated on poly-ornithine-laminin-coated plates for neural differentiation in 0.5x N2 and B27 for two more weeks. Monolayer differentiation of Human iPSCs was carried out as described (Espuny-Camacho et al., 2013) with slight modification. Single-cell suspension of human iPSCs was plated at low density in the presence of 10 μM ROCK inhibitor (Selleckchem) and treated with 125 nM dorsomorphin (Tocris) from day 2 to day 18. After cells reached confluency, single cells were re-plated on a polyornithine-laminin or Matrigel-coated plate and cultured for another 6–10 weeks in 0.5x N2 and 0.5x B27 in a 1:1 mix of Neurobasal and Knockout D-MEM/F-12 medium (all Invitrogen).

### Immunocytochemistry and image analysis

For immunocytochemistry (ICC), cells were fixed in 4% (wt./vol.) paraformaldehyde (Affymetrix # 19943) and 4% (wt./vol.) glucose (Sigma) in PBS for 10 min at 37°C and then permeabilized in blocking solution with 5% (vol./vol.) donkey serum (Jackson Immunoresearch # 017-000-121), 3% (wt./vol.) BSA (Sigma # A9647) and 0.1% Triton X-100 (Sigma) in DPBS (Corning) for 1 h at room temperature. Cells were incubated overnight with primary antibodies in a blocking solution at 4°C. Alexa-conjugated secondary antibodies (Life Technologies) were added to the blocking solution for 1 hour at room temperature, followed by incubation of cells in DAPI (Affymetrix) for an additional 15 min. A list of antibodies has been provided (Supplementary Table S2). Visualization of neurons was on a Nikon Ti2 inverted microscope with a 10X, 20X, or ×40 objective. Pictures of cells cultured under different experimental conditions were taken with the same exposure time and contrast/brightness parameters. For quantification of nuclear markers (such as NeuN and Ki67), positive cells were counted and expressed as a percentage of total (DAPI+) cells. For the quantification of cytoplasmic markers (such as TUBB3, ALDH1L1, GFAP, and DCX), pictures were taken with the same exposure time and contrast/brightness parameters. The total area showing immunoreactivity for a particular marker was determined using Fiji and normalized to the total area positive for DAPI, which estimates the total number of cells present in a given field. A minimum of three random fields containing at least 100 cells were analyzed for each condition. Fiji (NIH) was used to calculate scale bars for each image. Unless stated otherwise, values are shown as mean ± SEM, and asterisks in figures denote significance from Student’s t-test between two groups. A total of three independent experiments were performed using the same batch of iPSCs (*N* = 3). For all figures, *p < 0.05, **p < 0.01, ***p < 0.001, n.s. = non-significant.

### Flow cytometry analysis

At indicated time points, cells were collected using Accutase (Innovative Cell Technologies), fixed in 0.1% paraformaldehyde, and permeabilized in 90% methanol before being filtered through a 70 μm-cell strainer. The dissociated cells were resuspended in FACS buffer comprised of PBS, 2% donkey serum, and 0.01% NaN3. Cells were incubated overnight at 4°C with primary antibodies or isotype rabbit IgG, followed by washing and staining with secondary antibodies for 1 h. For cell cycle analysis, BrdU (10μM; BD Biosciences) was added to the cell culture medium for 45 min. BrdU-specific antibody was applied after membrane permeabilization according to the manufacturer’s manual, followed by DAPI staining and flow analysis. Samples were analyzed with a FACS Calibur (BD Biosciences) running FlowJo software (Treestar).

### QRT-PCR, RNAseq, and data analysis

Total RNA was collected using TRIzol reagent (Life Technologies) and purified using RNeasy mini Kit (Qiagen) using the manufacturer’s instructions. RNA quality was checked using Tapestation 2,200 (Agilent Technologies) and quantified using the Qubit instrument (Life technologies). Reverse transcription was done using a High-Capacity cDNA Reverse Transcription Kit (Applied Biosystems #4368814). QRT-PCR was performed using PowerUp SYBR Green Master Mix (Life technologies # A25741) with ABI PRISM 7300 sequence detection system. Primers for NFIB (PPH06907A), POU3F4 (PPH07129B), SOX11 (PPH20114A) and GAPDH (PPH00150F) were purchased from Qiagen (Catalogue # 330001). TrueSeq stranded mRNAseq libraries were prepared from 1 μg of total RNA (Illumina mRNAseq kit, RS-122-2,103) and sequenced using Illumina HiSeq 2500 PE-100. Experiments were performed in triplicate. Sequencing results were uploaded to Illumina BaseSpace for mapping (BaseSpace App v1.0, TopHat v2) and differential gene analysis (BaseSpace App v1. 1, CuffLinks v2.1.1). As described previously (Do et al., 2015), we performed Principal component analysis (PCA) on whole transcriptome (bulk RNAseq) samples from DS and 2N iPSCs, comparing quantile normalized FPKMs of gene expression across all samples and replicates. We utilized PCA as an unsupervised learning method for clustering as a quality control step to ensure the sequenced samples and replicates have similar gene expression profiles. The mRNAseq data for cortical progenitor cells (CPCs) shown in Figure 6 was analyzed using Basepair software (http://www.basepairtech.com/) with pipelines including the following steps. Raw reads were aligned to the transcriptome derived from UCSC genome assembly hg19, using STAR (Dobin et al., 2013) with default parameters. Read counts for each transcript were measured using feature Counts (Liao et al., 2014). Differentially expressed genes were determined using DESeq2 (Love et al., 2014) and a cut-off of 0.05 on adjusted *p*-value (corrected for multiple hypotheses testing) was used for creating lists and heatmaps unless otherwise stated. Data were further analyzed using QIAGEN’s Ingenuity® Pathway Analysis (IPA®, QIAGEN Redwood City, www.qiagen.com/ingenuity). The networks, functional analyses, etc., were generated using QIAGEN’s Ingenuity Pathway Analysis (IPA®, QIAGEN Redwood City, www.qiagen.com/ingenuity). Gene set enrichment analysis of the mRNAseq data was performed with GSEA, Broad Institute (Mootha et al., 2003; Subramanian et al., 2005).

### Chromatin immunoprecipitation, high-throughput sequencing, and data analysis

ChIPseq was performed as previously described (Bernt et al., 2011; Deshpande et al., 2014). Cell samples were cross-linked with 1% formaldehyde for 10 min and then stopped by adding glycine to the final concentration of 125 mM. The fixed cells were lysed in SDS buffer, dounce homogenized, and the chromatin was fragmented using sonication. Sheared chromatin was incubated with the indicated antibody and recovered by binding to protein A/G agarose (Millipore). Eluted DNA fragments were used for library preparation. Libraries were prepared using the NEBNext Ultra II DNA Library Prep Kit for Illumina from NEB according to the manufacturer’s instructions. Briefly, DNA was end-repaired, phosphorylated and dA tailed prior to adaptor ligation with NEBNext adaptors. After adapter ligation, DNA was PCR amplified with NEBNext primers for Illumina for 15 cycles, and library fragments of ∼250bp (insert plus adaptor and PCR primer sequences) were size selected using Ampure Beads (Becton Dickinson), followed by DNA cleanup using standard magnetic bead-based DNA purification protocols. The purified DNA was captured on an Illumina flow cell for cluster generation. Libraries were sequenced on the NextSeq500 following the manufacturer’s protocols. For the Pax6 ChIPseq dataset, high-throughput reads were aligned to the human genome assembly hg19 using Bowtie for Illumina (Galaxy Version 1.1.2) (Langmead et al., 2009). Binding sites were identified with MACS2 (Galaxy Version 2.1.0.20151222.0) (Zhang et al., 2008) and were assigned to the nearest RefSeq transcription start site within 3 kb using R package ChIPpeakAnno (Zhu et al., 2010; Zhu, 2013). ChIP peak comparison and visualization were made using the R package ChIPseeker (Yu et al., 2015) Binding heat maps were created using bed files. Gene ontology and disease ontology were performed on the annotated binding sites using the R package cluster profile (Yu et al., 2012).

### Statistical analysis

Statistical analysis was performed using Graph Pad Prism Software, San Diego, CA, United States, with an unpaired Student’s t-test. The data are shown as mean ± SEM from three independent experiments. *p* values of <0.05 were considered statistically significant. Graphs were also designed using Graph Pad Prism software.

## Results

### Generation of induced pluripotent stem cells from a mouse model of down syndrome

**FIGURE 1:**
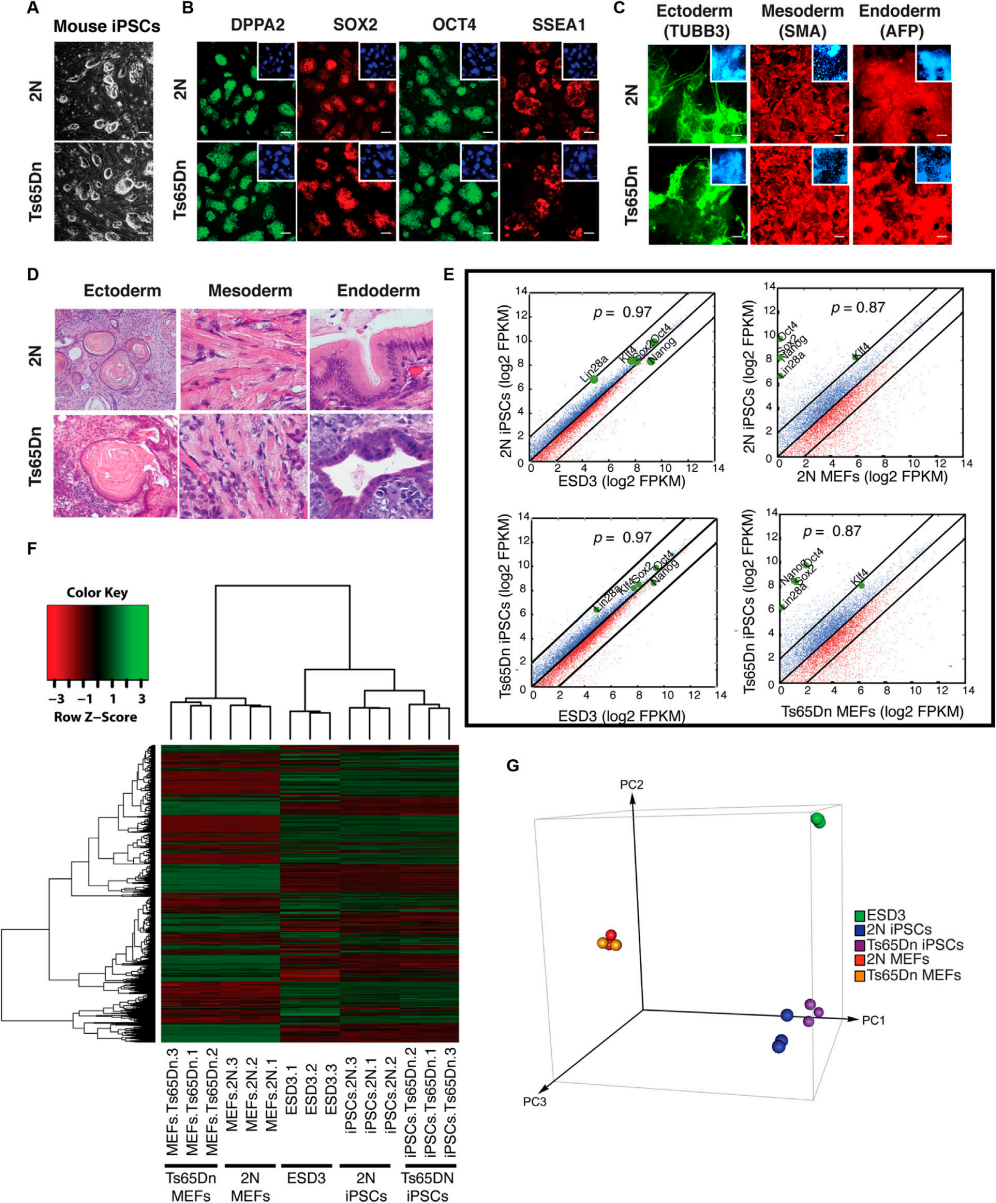
Mouse iPSCs generation and characterization. **(A)** Representative images of 2N and Ts65Dn mouse iPSCs (miPSCs) clones on the feeder layer. **(B)** Pluripotency characterization of miPSCs using ICC for pluripotency-associated markers DPPA2 (green), SOX2 (red), OCT4 (green), and SSEA1 (red). Inserts show corresponding DAPI (blue) staining for each image. **(C)** *In vitro* developmental potential of 2N and Ts65Dn miPSCs is characterized by the expression of markers of three germ layers. ICC was done for Tubulin beta 3 (TUBB3) (green) for ectoderm, Smooth muscle actin (SMA) (red) for mesoderm, and Alpha fetal protein (AFP) (red) for endoderm. Inserts show corresponding DAPI (blue) staining for each image. **(D)** Representative images of ectodermal (skin epithelium), mesodermal (muscle), and endodermal (cuboidal epithelium) lineage tissues in teratomas were used to assess *in vivo* developmental potential of 2N and Ts65Dn miPSCs. **(E)** Pairwise correlation plots of the global gene expression of ESD3 with 2N miPSCs, ESD3 with Ts65Dn miPSCs, 2N MEFs with 2N miPSCs, Ts65Dn MEFs with Ts65Dn miPSCs. The black line indicates log2 two-fold changes in gene expression levels between the paired cell types. Upregulated genes in ordinate samples compared with abscissa samples are shown in blue; those downregulated are shown in red. The positions of pluripotent genes (*Pou5f1/Oct4*, *Sox2*, *Nanog*, *Klf4*, and *Lin28*) are shown as green dots. The gene expression levels are in the log_2_ scale. **(F)** Heat map of global gene expression. On the top is depicted the gene expression color key. Samples labels are shown at the bottom. **(G)** Principal component analysis (PCA) of global gene expression among replicates from the 2N MEFs, 2N miPSCs, Ts65Dn MEFs, Ts65Dn miPSCs, and ESD3.

To develop studies in mouse models of DS and DS human iPSCs, we generated mouse iPSCs using Ts65Dn and 2N mouse embryonic fibroblasts (MEFs) isolated from E18.5 mouse embryos. To generate mouse-induced pluripotent cells, MEFs were reprogrammed using retroviral overexpression of OCT4, SOX2, KLF4, and *c-*MYC (OSKM). Individual miPSCs clones were isolated and expanded (Figure 1A). 2N and Ts65Dn miPSCs expressed pluripotency markers DPPA2, SOX2, OCT4, and SSEA1 (Figure 1B). Differentiating 2N and Ts65Dn cells expressed Tubulin beta 3 (TUBB3), a marker of ectoderm, Smooth muscle actin (SMA), a marker of mesoderm, and Alpha fetal protein (AFP), a marker of endoderm, thus providing evidence of the *in vitro* developmental potential of both 2N and Ts65Dn miPSCs (Figure 1C). The *in vivo* developmental potential of 2N and Ts65Dn miPSCs was tested by injecting miPSCs into SCID mice. Teratoma formation was evident in the presence of ectoderm (skin), mesoderm (muscle), and endoderm (cuboidal epithelium) for both 2N and Ts65Dn miPSCs (Figure 1D). Whole transcriptome analysis of 2N and Ts65Dn MEFs and miPSCs was performed to confirm pluripotency; mouse embryonic stem cells (ESD3) were included as a control. Pairwise correlation plot analysis of mRNA expression data demonstrated that 2N miPSCs and Ts65Dn miPSCs were very similar to ESD3; note the very similar values for the mRNAs of the pluripotency genes *Lin28A, Klf4, Sox2, Oct4, Nanog* (Spearman coefficient for all mRNAs: 2N miPSCs vs. ESD3 *p* = 0.97, Ts65Dn miPSCs vs. ESD3 *p* = 0.97). As expected, the miPSCs differed significantly from their corresponding MEFs; note the very distinct values for *Lin28A, Klf4, Sox2, Oct4,* and *Nanog* (Spearman coefficient for all mRNAs: 2N miPSCs vs. 2N MEFs *p* = 0.87 and Ts65Dn MEFS vs. Ts65Dn miPSCs *p* = 0.87) (Figure 1E). Heat map analysis of these data showed that the transcriptomic profile of Ts65Dn miPSCs and 2N miPSCs was very similar to pluripotent mouse ESD3 cells and significantly different from their MEFs (Figure 1F). Finally, using principal component analysis, the first principal component discriminated 2N MEFs and Ts65Dn MEFs from 2N iPSCs and Ts65Dn iPSCs. Not surprisingly, PC1 modestly discriminated between 2N miPSCs and Ts65Dn miPSCs; ESD3 differed by second principal component (PC2) (Figure 1G). We conclude that both 2N and Ts65DN miPSCs were pluripotent.

### Ts65Dn miPSCs displayed reduced neural differentiation and increased astroglial differentiation

**FIGURE 2:**
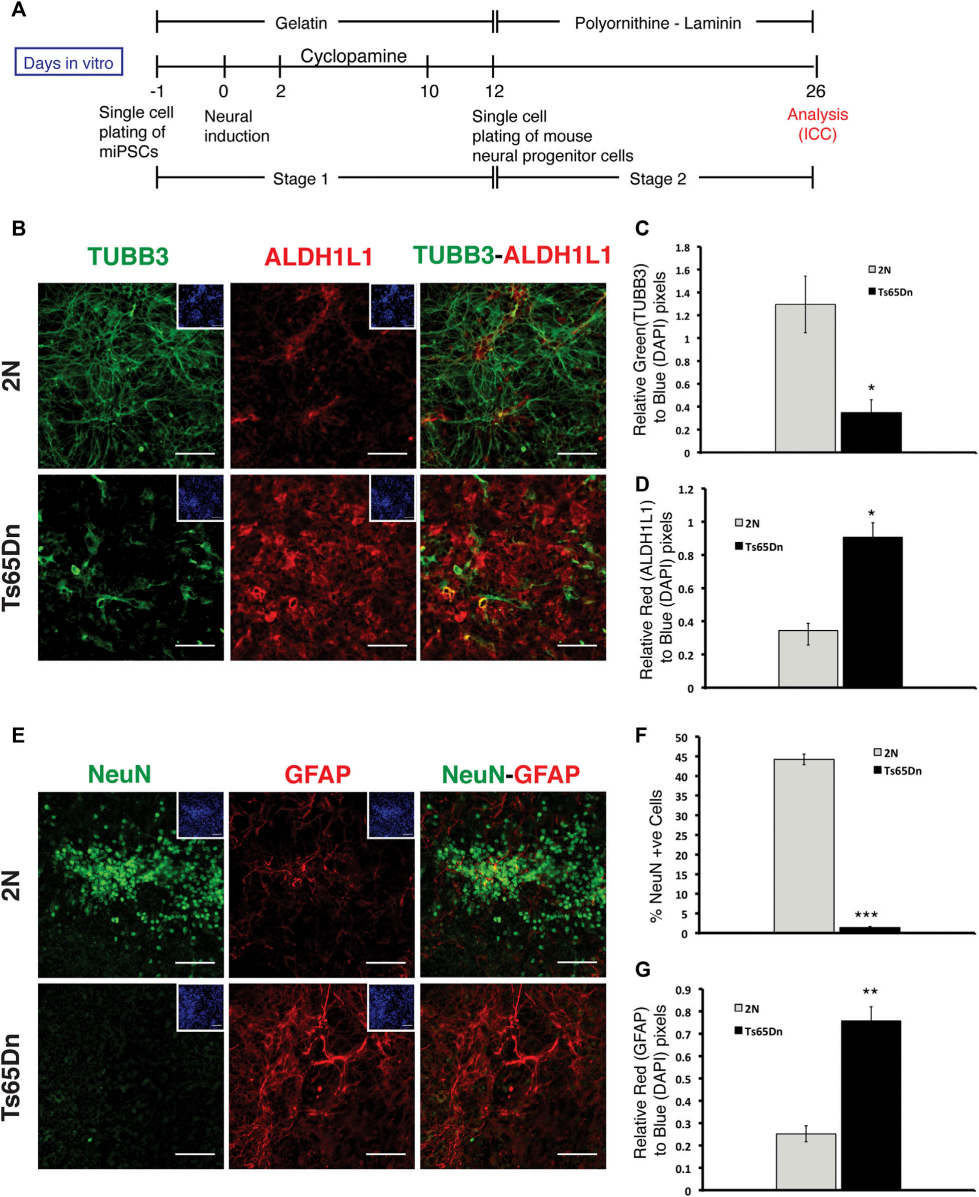
Analysis of monolayer-mediated neural differentiation of 2N and Ts65Dn miPSCs. **(A)** Schematic representation of neural differentiation protocol for miPSCs. Stage 1 is defined as the differentiation of iPSCs to neural progenitors, and stage 2 is defined as the differentiation of neural progenitors to postmitotic neural differentiation. **(B)** Representative images of TUBB3 (green) and ALDH1L1 (red) expressing cells and overlays of TUBB3 and ALDH1L1 images at DIV 26. Corresponding DAPI (blue) images have been shown as inserts **(C)** Quantification of TUBB3 expression in 2N and Ts65Dn cells normalized by DAPI staining. **(D)** Quantification of ALDH1L1 expression in 2N and Ts65Dn cells normalized by DAPI staining. **(E)** Representative images of NeuN (green) and GFAP (red) expressing cells and overlays of NeuN and GFAP images. Corresponding DAPI (blue) images have been shown as inserts. **(F)** Quantification of NeuN + nuclei in 2N and Ts65Dn cells against a total number of nuclei identified by DAPI staining. **(G)** Quantification of GFAP expression in 2N and Ts65Dn cells normalized by DAPI staining. Data are represented as mean ± SEM. A total of three independent experiments were performed using the same batch of iPSCs (*N* = 3). *p < 0.05, **p < 0.01, ***p < 0.001, n.s. non-significant. The scale bar is 50 µm. (See also Supplementary Figure S1).

The embryoid body (EB) method was utilized to induce neural differentiation of 2N and Ts65Dn miPSCs. 2N and Ts65Dn miPSCs generated EBs within 3 days of initiating EB formation. EBs plated onto poly D-lysine-laminin-coated dishes were treated with retinoic acid to induce neuronal differentiation, as marked by TUBB3 and MAP2 immunostaining. Compared to EBs generated from 2N miPSCs, EBs from Ts65Dn miPSCs displayed fewer TUBB3+ and MAP2+ processes (Supplementary Figure S1). EBs based methods suffer from the formation of local patterning centers whose impact is difficult to assess, in addition to difficulty quantifying neurogenesis. Thus, to overcome these difficulties in assessing whether or not the reduction in neuronal processes was due to a decrease in neuronal differentiation, we used a previously described monolayer-based method in which cortical neural differentiation relies on the intrinsic properties of pluripotent cells (Gaspard et al., 2008; Gaspard et al., 2009). This method consists of two stages. In stage 1, iPSCs are differentiated towards the generation of cortical neural progenitors (neurogenic stage), and in stage 2, cortical neural progenitors are differentiated towards postmitotic neurons (neural differentiation stage). Single-cell suspension of iPSCs is plated to generate a monolayer. After 24 h, neural induction is initiated by the withdrawal of leukemia inhibitory factor (LIF). On days 2 through 10, the Sonic hedgehog (SHH) inhibitor Cyclopamine is added to enhance forebrain identity. On day 12, stage 2 is initiated by creating single cell suspensions followed by re-plating cells on polyornithine-laminin coated plates; differentiation then proceeds for another 2 weeks (Figure 2A). At the end of phase 2, Ts65Dn and 2N cells were stained with TUBB3. While robust TUBB3 staining was present in 2N cultures, Ts65Dn culture showed significantly reduced TUBB3 staining. (Figure 2B). Quantitative analysis of TUBB3 staining revealed a ∼3-fold reduction in Ts65Dn cultures compared to 2N cultures (Figures 2B,C). In contrast to the results for TUBB3, staining for ALDH1L1, a marker for astrocytes, was significantly increased in Ts65Dn cultures compared to 2N cultures (Figures 2B,D).

To further explore and confirm the apparent decrease in neuronal differentiation in Ts65Dn cultures, we immunostained 2N and Ts65Dn cells for NeuN, a nuclear marker for neurons, which emerges during early neurogenesis in postmitotic neurons and remains in differentiating and terminally differentiated neurons (Gusel’nikova and Korzhevskiy, 2015). Quantitative analysis of NeuN-expressing cells revealed a ∼9-fold reduction in the number of neurons in Ts65Dn compared to 2N cultures (Figures 2E,F). On the contrary, GFAP + cells showed a ∼4-fold increase in Ts65Dn cells compared to 2N cells (Figures 2E,G). We conclude that Ts65Dn miPSCs give rise to fewer neurons and more glial cells than 2N miPSCs, which is consistent with previous *in vivo* studies in Ts65Dn mice (Contestabile et al., 2007; Contestabile et al., 2009), indicating the robustness of *in vitro* neurogenesis model using iPSCs.

### Ts65Dn cells display aberrant cell cycle regulation during neuronal differentiation

**FIGURE 3:**
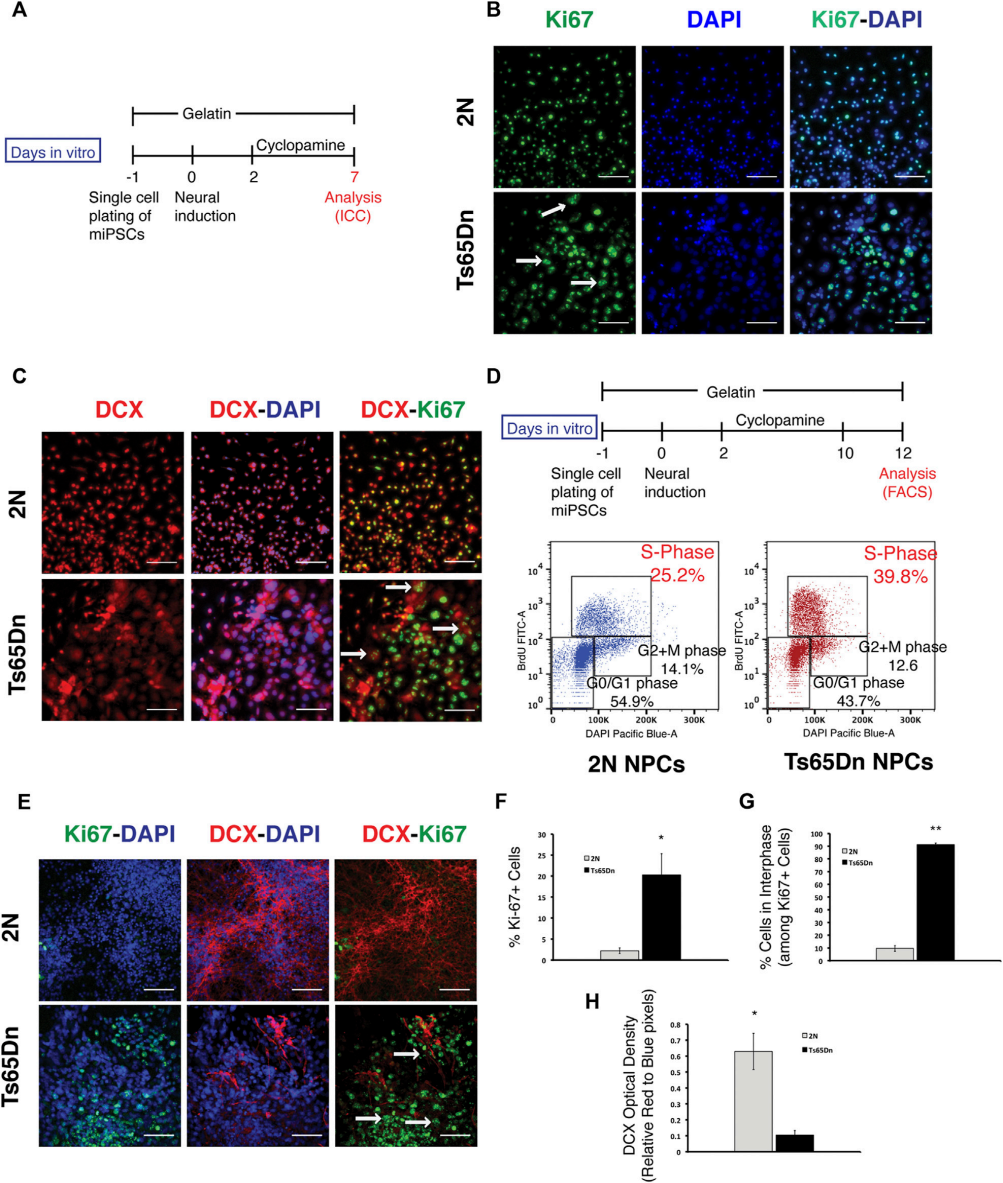
Analysis of mouse progenitor cells during neural differentiation. **(A)** Schematic representation of progenitor differentiation from mouse iPSCs. **(B)** Ki67 staining and its overlay with DAPI stained nuclei on DIV 7. Cells in the M phase of the cell cycle show whole nuclei expression, while cells in the interphase (G1/S/G2 phase) show a punctate pattern. Examples of Ki67 + cells with punctate expression patterns are shown with an arrow. **(C)** Progenitor cells were stained with Doublecortin (DCX) at DIV 7 of differentiation. Representative images showing DCX (red) expression. DCX-DAPI image overlay shows perinuclear staining of DCX in 2N cells and diffused staining in Ts65Dn cells. Overlay of DCX and Ki67 shows that cells with Ki67 punctate expression pattern have diffused DCX expression patterns, as indicated by an arrow. **(D)** Cells were labeled with BrdU on DIV 12 of differentiation. BrdU labeled cells were stained with anti-BrdU FITC antibody and DAPI before FACS analysis. The percentage of cells in each phase of the cell cycle is shown. **(E)** Representative images showing an overlay of Ki67 (green) overlaid with DAPI (blue), DCX (red) overlaid with DAPI (blue), and DCX (red) overlaid with Ki67(green) expressing cells. Cells with a Ki67 punctate pattern are shown with an arrow **(F)**. Quantification of Ki67 + nuclei in 2N and Ts65Dn cells against a total number of nuclei identified by DAPI staining and **(G)** quantification of Ki67 + cells with punctate expression against a total number of Ki67 + cells **(H)** Quantification of DCX expression in 2N and Ts65Dn cells normalized by DAPI staining. Data are represented as mean ± SEM, where three independent experiments were performed using the same batch of iPSCs (*N* = 3). *p < 0.05, **p < 0.01, ***p < 0.001, n.s. non-significant. The scale bar is 50 µm. (See also Supplementary Figure S2).

2N and Ts65Dn miPSCs were analyzed for apoptosis using Tunnel assay during the neurogenic stage of differentiation to understand the underlying cause of reduced neural differentiation in Ts65Dn cells. The Tunnel assay did not find increased apoptosis in Ts65Dn cells (Supplementary Figure S2A). Further, to check the relation between cell cycle and neural differentiation during the neurogenic stage, cells were analyzed with Ki67 to mark proliferating cells and Doublecortin (DCX), a microtubule-associated protein expressed by neuronal precursor cells and immature neurons and present in the perinuclear region (Francis et al., 1999; Brown et al., 2003) (Figure 3A). Ki67 immunostaining differed between 2N and Ts65Dn cells in the early stages following neural induction. Ki67 + nuclei in 2N cultures were uniformly small, and staining marked the entire nucleus, indicating that most of the 2N cells were in the M phase of the cell cycle (Verheijen et al., 1989). In contrast, in Ts65Dn cultures, Ki67 stained nuclei were large, often irregularly shaped, and staining was punctate. Punctate expression of Ki67 is indicative of cells in interphase (G1/S/G2 phase) (Verheijen et al., 1989) (Figure 3B). DCX immunostaining at an early stage also revealed differences between 2N and Ts65Dn cells. In 2N cultures, many small cells were uniformly and brightly stained. Ts65Dn cells, in contrast, demonstrated variability in immunopositivity for DCX; staining was typically diffuse and in many cells staining intensity was low (Figure 3C). Overlay of DAPI and DCX images showed that most nuclei were positive for both markers in 2N and Ts65Dn cultures; many Ts65Dn cells showed diffuse, low-intensity DCX staining in cells with large nuclei. Ki67 and DCX overlay images further informed the analysis. Overlapping staining was frequently present in 2N cells, suggesting that cells in the M phase express a marker of neuronal differentiation. In Ts65Dn cultures, cells that demonstrated low-intensity DCX staining typically showed punctate Ki67 staining, suggesting that this pattern of DCX staining characterizes cells in interphase.

To further examine the difference in cell cycle between 2N and Ts65Dn cells, BrdU was used to label cells at the late phase of the neurogenic stage and then stained with DAPI (Figure 3D). FACS analysis showed that ∼40% of Ts65Dn cells were in the S phase compared to ∼25% of 2N cells. FACS analysis also found that ∼55% of 2N cells were in G0/G1 phase compared to ∼44% of Ts65Dn cells in the G0/G1 phase of the cell cycle (Figure 3D). These data are evidence of dysregulation of the cell cycle in Ts65Dn cells, with more cells in the S phase and fewer in G0/G1 at the end of the neurogenic stage.

Further, to analyze the effect of cell cycle changes in the neurogenic stage (stage1) on neuronal differentiation, cells were examined using ICC for Ki67 and DCX at the end of stage 2 (Figure 2A). Although 2N cultures and Ts65Dn cultures showed a similar number of DAPI + cells (Supplementary Figure S2B), 2N cells showed little evidence for Ki67 staining, while in Ts65Dn cultures, many more cells were Ki67+ and demonstrated the punctate pattern. Quantitation showed that in Ts65Dn cultures, ∼20% of cells were Ki67+ (Figures 3E,F) and that ∼90% had punctate staining (Figure 3G). In contrast, 2N cells were only ∼2% Ki67+ (Figures 3E,F), and only ∼2% of these cells had punctate staining (Figure 3G). These data point to a relative deficit in the progression of Ts65Dn cells beyond the M phase, with many more remaining in the interphase. DCX immunostaining confirmed the relative neurogenic deficit in Ts65Dn cells. Whereas DCX staining in 2N cells was robust with dense staining of cell bodies and processes, in Ts65Dn cultures, staining was sparse and weak, and diffuse staining of many cells continued to be present. Quantitation of DCX optical density showed a ∼ 6-fold decrease in Ts65Dn cultures (Figures 3E,H). We conclude that Ts65Dn cells differ significantly from 2N cells in progression through the cell cycle.

### Human down syndrome iPSCs showed reduced neural differentiation

**FIGURE 4:**
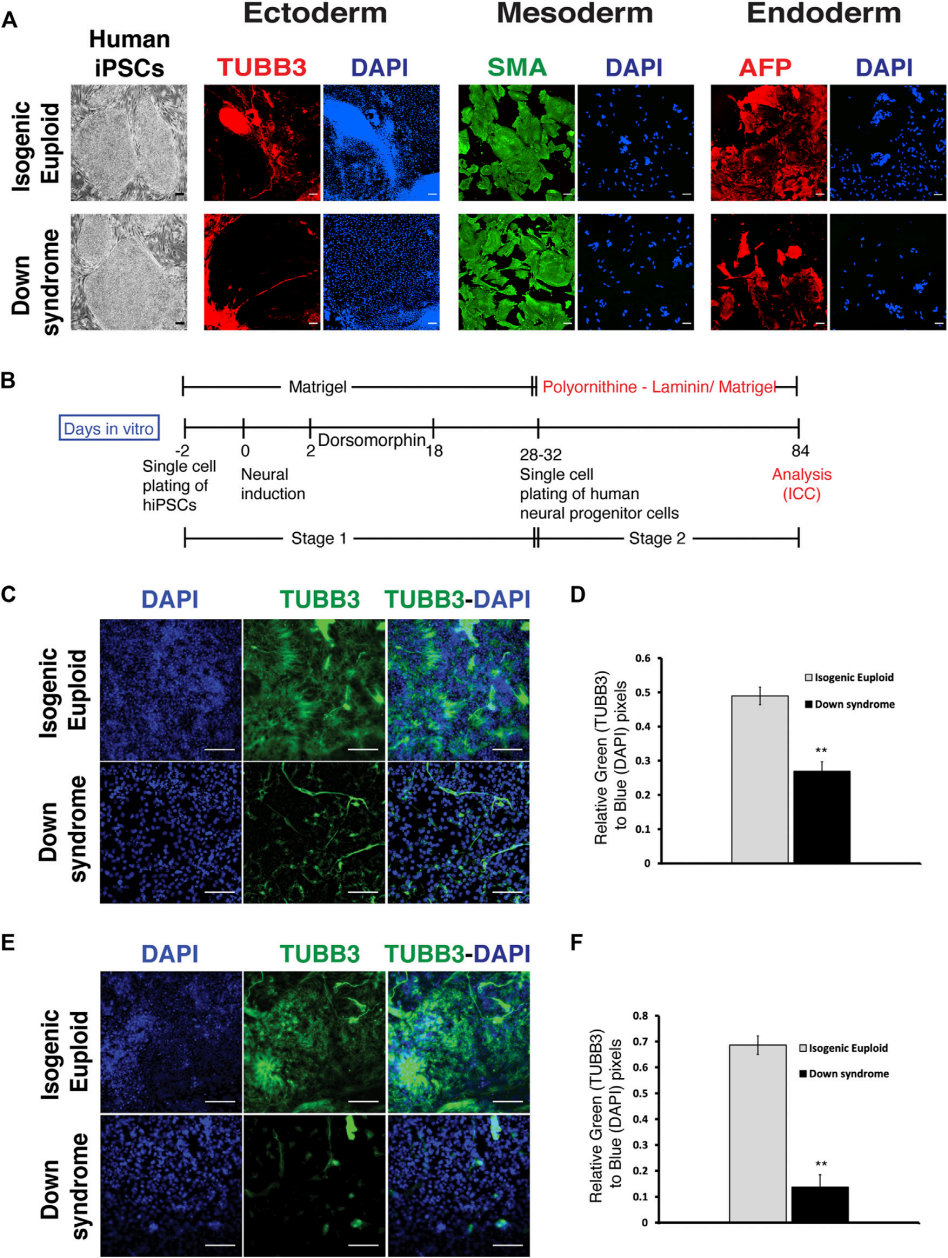
Monolayer-mediated differentiation of Down syndrome human iPSCs display reduced neural differentiation. **(A)** Representative images of Down syndrome human iPSCs (hiPSCs), its isogenic euploid hiPSCs, and three germ layer differentiation. Three germ layer analysis was done using ICC for Tubulin beta 3 (TUBB3) (red) for ectoderm, Smooth muscle actin (SMA) (green) for mesoderm, and Alpha fetal protein (AFP) (red) for endoderm. Corresponding DAPI (blue) staining for each image has been shown. Scalebar is 100 μM. **(B)** Schematics of neural differentiation protocol of hiPSCs on poly Ornithine-Laminin or Matrigel. Stage 1 is defined as the differentiation of iPSCs to neural progenitors, and stage 2 is defined as the differentiation of neural progenitors to postmitotic neural differentiation. **(C)** Representative images of TUBB3 (green) expressing human cells, corresponding DAPI images, and their overlay and quantification of TUBB3 expression normalized by DAPI staining **(D)** on poly Ornithine-Laminin. **(E)** Representative images of TUBB3 (green) expressing cells, corresponding DAPI images, and their overlay and quantification of TUBB3 expression normalized by DAPI staining **(F)** on Matrigel during phase 2. Data are represented as mean ± SEM A total of three independent experiments were performed using the same batch of iPSCs (N = 3). *p < 0.05, **p < 0.01, ***p < 0.001, n.s. non-significant. The scale bar is 50 µm. (See also Supplementary Figure S3).

To ask if the findings in the Ts65Dn model were also present in human DS cells, we used a similar protocol to explore neuronal differentiation in DS cells. For hiPSCs, we used a previously described isogenic pair of DS hiPSCs and isogenic euploid hiPSCs (Maclean et al., 2012). These human iPSCs were characterized for pluripotency by expression of pluripotency markers OCT4 and SOX2 using ICC and quantitative PCR for OCT4, SOX2, and NANOG. In addition, we also performed three germ layer differentiation and karyotyping analyses. Pluripotency marker analysis and three germ layer potential analyses confirmed pluripotency, and karyotyping analysis confirmed the expected karyotype of DS and its isogenic euploid hiPSCs (Figure 4A and Supplementary Figure S3). As for mouse iPSCs, the protocol for cortical neural differentiation of human iPSCs (Espuny-Camacho et al., 2013) also consisted of two stages that are stage 1 (neurogenic stage) and stage 2 (neural differentiation stage). Human iPSCs in single cell suspension were plated onto Matrigel-coated dishes before inducing differentiation by removing bFGF. Dorsomorphin, a BMP inhibitor, was added from days 2 through 18 to prevent non-ectodermal differentiation. For the differentiation of progenitor cells from stage 1, single-cell suspensions were re-plated at low density on either polyornithine-laminin or Matrigel (Figure 4B) for an additional 6–10 weeks to observe early neural differentiation.

Analysis performed at the end of stage 2 found a significant reduction of TUBB3+ neurons in DS cultures compared to those containing isogenic euploid cells (Figure 4C). Quantification found a ∼2-fold decrease in TUBB3+ neurons in DS culture (Figure 4D). To rule out the effect of substrate on neuronal differentiation, we repeated these studies on Matrigel and confirmed the decrease in TUBB3 immunostaining in DS cells (Figure 4E). Quantification found a ∼6-fold reduction in TUBB3+ neurons in DS culture (Figure 4F). Thus, DS cells show reduced neuronal differentiation as exhibited by DS fetal brain sections, Ts65Dn mice, and Ts65Dn iPSCs, indicating the robustness of the *in vitro* DS neurogenesis modeling carried out in this study.

### Human down syndrome cells displayed abnormal cell cycle during neural differentiation

**FIGURE 5:**
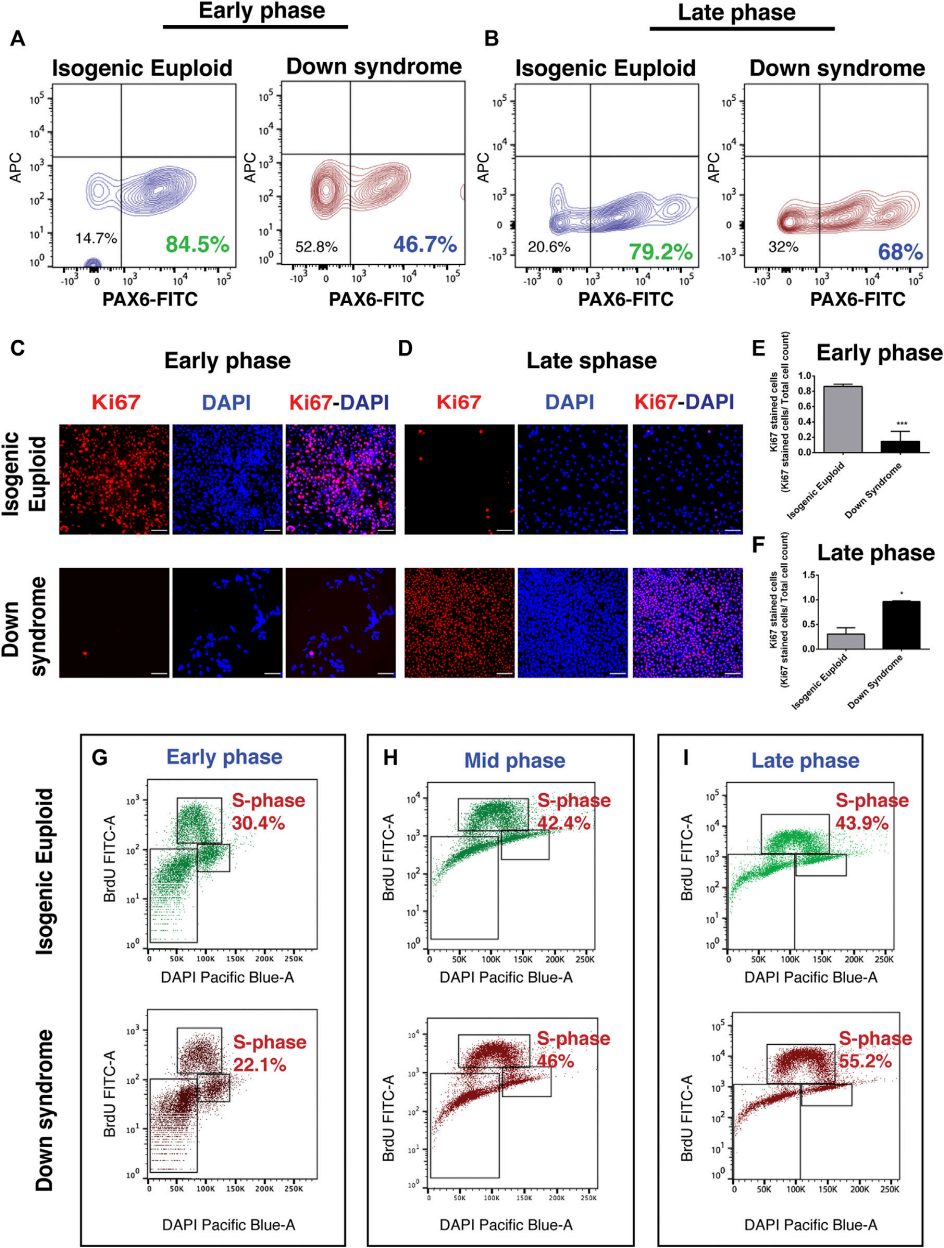
Analysis of human neural progenitor cells during neural differentiation. **(A)** FACS analysis at the early phase (DIV 18) of stage 1 for quantification of PAX6+ cells. The percentage of PAX6+ cells is shown. **(B)** FACS analysis at late phase (DIV 28) of stage 1 for quantification of PAX6+ cells. The percentage of PAX6+ cells is shown. **(C)** Representative image of Ki67 at the early phase (DIV 18) of stage 1 along with corresponding DAPI image and their overlay. **(D)** Representative image of Ki67 at late phase (DIV 28) of stage 1 along with corresponding DAPI image and their overlay. **(E–F)** Quantification of Ki67 + nuclei normalized by DAPI. Data are represented as mean ± SEM where *N* = 3. A total of three independent experiments were performed using the same batch of iPSCs. *p < 0.05, **p < 0.01, ***p < 0.001, n.s. non-significant. Scale bar is 100 μM. **(G–I)** Cells were labeled with BrdU on DIV10 **(G)**, DIV16 **(H)**, and DIV28 **(I)** and stained with anti-BrdU-FITC antibody and DAPI, and analyzed by FACS. The percentage of cells in each phase of the cell cycle is shown. (See also Supplementary Figure S4).

Our analysis using miPSCs found an increased number of S phase cells in Ts65Dn compared to its control 2N cells in the late phase of the neurogenic stage. In addition, Chakrabarti et al. also found an increased number of neural progenitor cells in Ts65Dn mouse at E16.5 compared to earlier stages (Chakrabarti et al., 2007). Thus, to investigate if DS hiPSCs also generate more neural progenitor cells in the late phase during the neurogenic stage, the generation of PAX6+ cells in an early and late phase of stage 1 was analyzed. PAX6 is a neuroectoderm marker implicated in regulating cell cycle length and cell cycle exit (Sansom et al., 2009) as well as in cell fate decisions (Zhang et al., 2010). Quantifying PAX6+ cells using FACS analysis at an early phase showed that ∼85% of isogenic euploid cells were PAX6+ compared to ∼47% of DS cells (Figure 5A). Repeating the analysis at a late phase showed that ∼79% of isogenic euploid cells were PAX6+ while ∼68% of DS cells were positive (Figure 5B). These results indicated that PAX6+ cell generation in DS compared to its euploid counterpart is initially reduced but later recovered, as shown by an increased number of PAX6+ cells at a late phase of the neurogenic stage.

To explore the cause of delayed PAX6+ neural progenitor generation in DS, the cell cycle during the neurogenic stage of neurogenesis was analyzed using Ki67 immunostaining. During the early neurogenic phase, most isogenic euploid cells were Ki67+, indicating the presence of cells in the proliferative phase. In contrast, fewer DS cells were immunopositive for Ki67 (Figure 5C and Supplementary Figure S4A). Quantitative analysis suggested a ∼4-fold reduction of Ki67 + cells in DS with a ∼2-fold reduction in DAPI + cells (Figure 5E and Supplementary Figure S4B). These results suggest that DS cells were predominantly present in G0 at an early phase of the neurogenic stage.

Further analysis at a late phase revealed that most isogenic euploid cells do not show Ki67 staining (Figure 5D and Supplementary Figure S4A), indicating that these cells have exited the cell cycle. On the contrary, most DS cells were still Ki67+ (Figure 5D and Supplementary Figure S4A), indicating the presence of cycling progenitor cells. Quantitative analysis suggested a ∼5-fold increase of Ki67 + cells in DS cultures and a ∼3-fold increase in DAPI + cells (Figure 5F and Supplementary Figure S4B).

Further, isogenic euploid and DS cells were subjected to BrdU pulse labeling to explore further deviations of DS cells from the normal cell cycle during the neurogenic stage of differentiation. FACS analysis at an early neurogenic stage showed that ∼30% of isogenic euploid cells were in the S-phase compared to ∼22% of DS cells (Figure 5G). At the middle of the neurogenic stage, ∼42% of isogenic euploid cells were in the S-phase *versus* ∼46% of DS cells (Figure 5H). At a late neurogenic stage, ∼44% of isogenic euploid cells in the S-phase *versus* ∼55% of DS cells (Figure 5I). BrdU pulse labeling analysis using FACS found ∼22% of DS cells in S-phase while a very small number of cells were Ki67 + at an early phase. Similarly, during the late phase, ∼43% of isogenic euploid cells were in S-phase, but a very small number could be detected by Ki67 staining. Despite this difference observed from these methods, both data indicate the reduced proliferation of DS cells at the early phase and increased proliferation of DS cells in the late phase. Summarizing the differences and their changes, decreased S phase occupancy by DS cells during the early phase in the neurogenic stage is not only reversed but reaches levels higher than those in isogenic euploid cells at later times. The changes point to marked differences in the timing of cell cycle events in the overall populations of isogenic and DS cells.

Thus, the analysis points to significant deviations from the cell cycle in DS cells. Several differences were apparent: 1) reduced proliferation of DS cells during the early phase of neurogenic stages, and 2) increased proliferation of DS cells during the late phase of the neurogenic stage. Both changes can be readily interpreted as decreasing the overall neurogenic potential of DS cells. In the first case, early cell divisions that support the creation of a larger neural precursor pool are curtailed. In the second, the persistence of cells in the S-phase may result in the delayed exit of progenitors from the cell cycle and fewer postmitotic neurons in the DS brain. Delayed exit from the cell cycle may also account for increased glial production, as proposed in the cell cycle hypothesis. A cell cycle hypothesis (Gotz and Huttner, 2005) proposes that time is a crucial factor, where actions of extrinsic or intrinsic factors depend on their duration of action. Thus, unrestricted progenitors need to exit the cell cycle in a timely fashion to commit toward neural lineage. Delayed exit from the cell cycle may also help explain delayed cortical lamination (Golden and Hyman, 1994) and recovery of the number of neurons observed in the DS visual cortex (Wisniewski et al., 1984).

### Dysregulated signaling pathways and gene expression in DS cells are correlated with reduced neurogenesis

**FIGURE 6:**
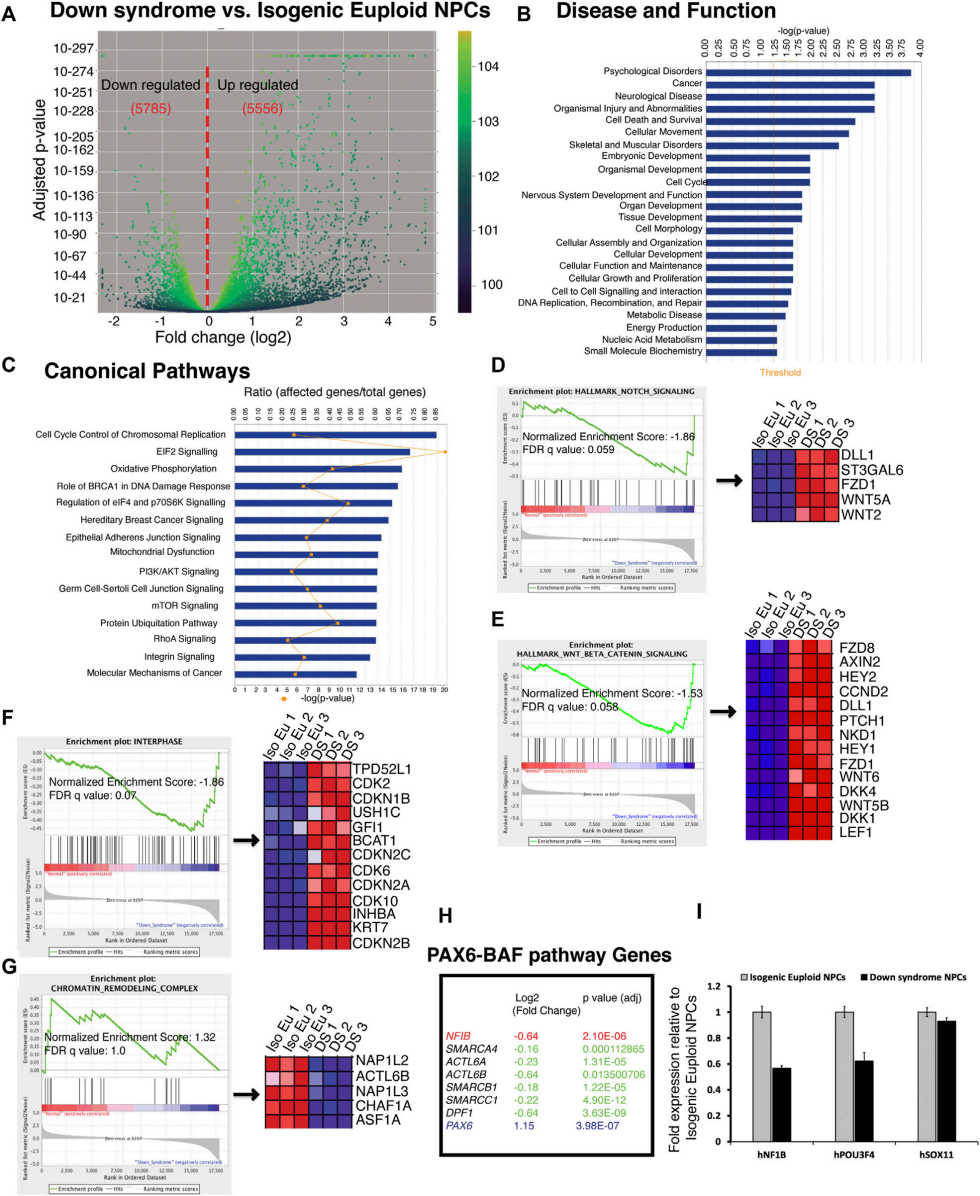
RNAseq analysis of global gene expression of Down syndrome and isogenic euploid DIV 24 progenitor cells. **(A)** Volcano plot shows downregulated and upregulated genes in Down syndrome cells compared to isogenic euploid cells. The number of genes is shown in red. **(B)** Top disease and functions term found through Ingenuity Pathway Analysis (IPA) with differentially expressed gene set in DIV 24 DS cells. The bar chart’s threshold line (orange) represents a *p*-value of 1.3. **(C)** Significant canonical pathways are involved in the well-characterized cell signaling and metabolic pathways associated with the differentially expressed genes in DIV 24 Down syndrome cells. The ratio (r) is calculated by the number of genes from the data set of the differentially expressed gene set that participate in a Canonical Pathway and dividing it by the total number of genes in that canonical pathway in IPA analysis. **(D,E)** Enrichment of Notch **(D)** and Wnt **(E)** signaling pathways using hallmark gene set analysis in Gene Set Enrichment Analysis (GSEA). Core genes enriched for indicated pathways are shown on the right. **(F,G)** Enrichment of Interphase **(F)** and Chromatin remodeling pathways **(G)** using gene ontology (GO) gene set analysis in GSEA. Core genes enriched for indicated pathways are shown on the right. FDR, false discovery rate. **(H)** List of selected genes of PAX6-BAF downstream pathway and downregulated genes of BAF complex. **(I)** QRT-PCR analysis of PAX6-BAF pathway target genes *NFIB, POU3F4,* and *SOX11.* Transcript levels were normalized to GAPDH expression. (See also Supplementary Figure S5).

We found in our analysis that DS-impaired neurogenesis is due to a biphasic defect in the cell cycle. While reduced proliferation has been described previously (Jiang et al., 2013; Hibaoui et al., 2014), we found evidence for the increased proliferation of DS NPC at the end of stage1. Thus, to explore the mechanism(s) responsible for the increased proliferation of DS neural progenitor cells, DS and isogenic late-phase progenitor cells were analyzed at the end of the neurogenic stage using whole transcriptome RNAseq. Volcano plot analysis showed that the gene expression pattern in DS cells differed significantly from isogenic euploid cells (Figure 6A). About 11,000 transcripts showed differences in DS *versus* isogenic euploid cells. DS cells showed both increase (5,556) and a decrease (5,785) in gene expression compared to isogenic euploid cells. Ingenuity Pathway Analysis (IPA) detected patterns consistent with various disorders, including psychological disorders, neurological disease, embryonic development, cell cycle, and nervous system development and function (Figure 6B). Canonical Pathway analysis identified the top three terms of cell cycle control of chromosomal replication, EIF2 signaling, and oxidative phosphorylation (Figure 6C). Gene Set Enrichment Analysis (GSEA) identified Notch signaling and Wnt/β-Catenin signaling as upregulated in DS cells and increased levels of the transcripts for *DLL1, FZD1, HEY1, HEY2, CCND2* (Figure 6D and Figure 6E). Other genes of interest included *SOX9* and *NFIA* in the Notch pathway and *REST*, a negative regulator of neurogenesis (Supplementary Table S1). GSEA analysis also found upregulation of interferon-alpha, interferon-gamma, and inflammatory pathways (Supplementary Figure S5), consistent with previous reports (Sullivan et al., 2016). Notably, the oxidative phosphorylation pathway in DS cells was downregulated compared to isogenic cells (Supplementary Figure S5). Consistent with cellular phenotypes, GSEA analysis using GO terms identified the interphase pathway as upregulated with enrichment of genes such as *CDK2* and *CDK6* in DS cells (Figure 6F). In addition, cell cycle regulators such as *CDCA3* and *E2F8* were upregulated, and *RB1*, *GEMININ,* and *CDT1* were downregulated (Supplementary Table S1). GO analysis found downregulation of chromatin remodeling pathway, with genes such as *NAP1L2* and *ACTL6B* (*BAF53B*), downregulated in DS cells (Figure 6G). In addition to *BAF53B*, several genes of the BAF complex such as *SMARCA4 (BRG1), ACTL6A (BAF53A)*, *SMARCB1 (INI1)*, *SMARCC1 (BAF155),* and *DPF1* were downregulated (Figure 6H). Considering the reported role of the PAX6-BAF interaction in neurogenesis (Ninkovic et al., 2013), we checked the expression levels of downstream genes *POU3F4, NFIB, and SOX11.* RNAseq data showed significant downregulation of *NFIB* expression (Figure 6H). QRT-PCR analysis confirmed the downregulation of *NFIB* and *POU3F4* (*BRN4*) (Figure 6I). This analysis demonstrates gene expression changes that may underlie changes in cell cycle regulation and deficits in neurogenesis seen in DS cells.

### PAX6 recruitment is affected in down syndrome progenitor cells

**FIGURE 7:**
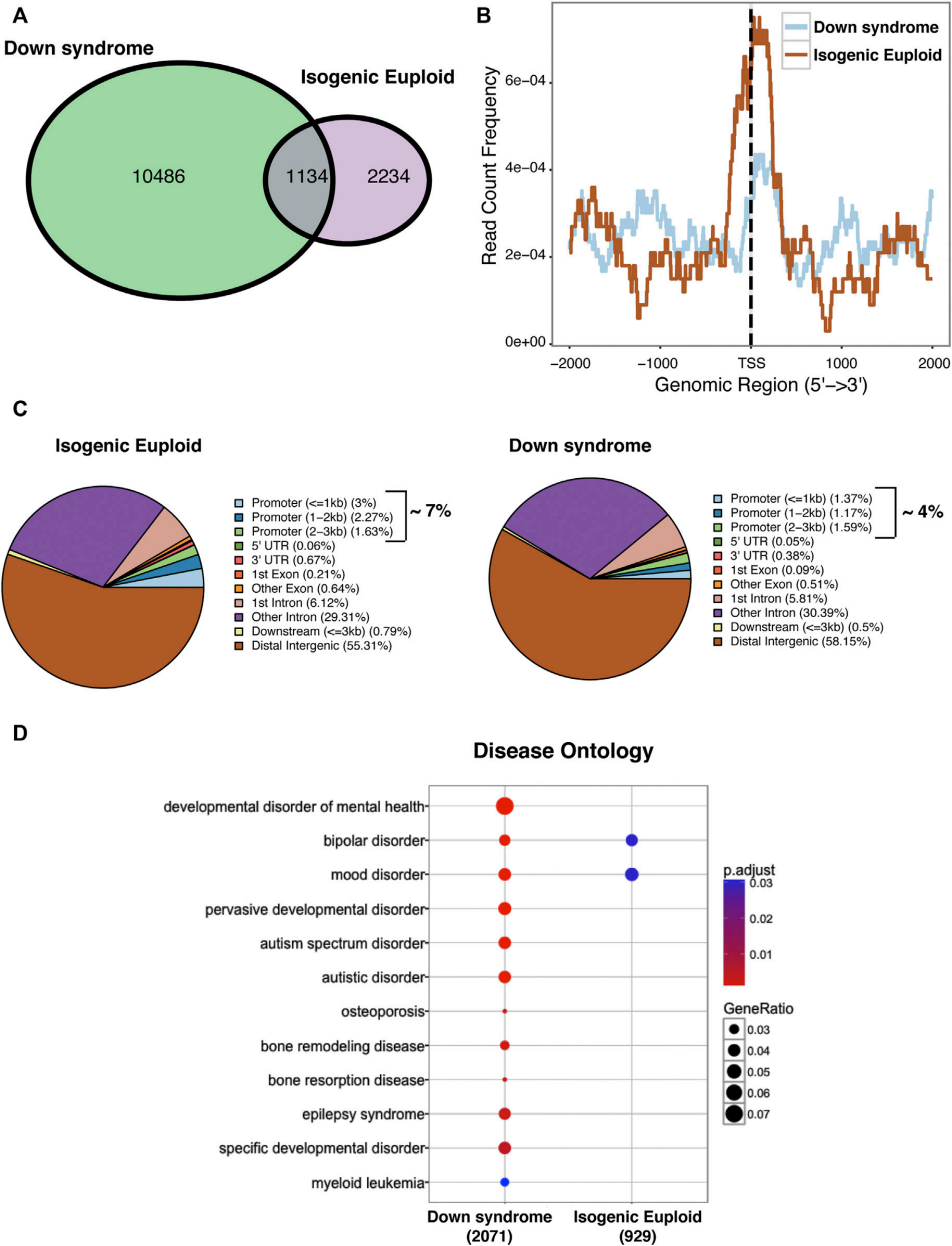
ChIPseq analysis of global PAX6 binding in DIV 24 Down syndrome and isogenic euploid cells. **(A)** Venn diagram showing the distribution of PAX6 binding sites in DS and isogenic euploid cells. **(B)** PAX6 binding profile in Down syndrome and isogenic euploid cells. **(C)** Pie chart analysis showing PAX6 binding on various genomic regions in DS and isogenic euploid cells. **(D)** Disease ontology analysis using PAX6 binding sites. (See also Supplementary Figure S6).

To pursue further a role for PAX6 in defective neurogenesis detected in DS cells, we examined the binding of this transcription factor at a late phase of stage 1 in DS and isogenic euploid cells using chromatin immunoprecipitation followed by high-throughput sequencing (ChIPseq). As shown in the Venn diagram, DS cells showed an overall increased binding of PAX6 compared to isogenic euploid progenitors. While 1134 PAX6 binding sites overlapped in DS and isogenic euploid progenitors, PAX6 bound to an additional 10486 unique sites in DS cells. In contrast, 2,234 binding sites occupied in isogenic cells were not occupied by PAX6 in DS cells (Figure 7A). Notably, there was a remarkable change in PAX6 binding at promoter-associated regions in DS cells compared with isogenic cells with decreased binding at the transcriptional start site in DS cells (Figure 7B). PAX6 was bound to 7% of promoter regions in isogenic cells *versus* 4% in DS cells. In contrast, distal intergenic regions in DS progenitors showed a slight increase in PAX6 binding (Figure 7C). Gene Ontology analysis of PAX6 binding sites detected differences in several genes associated with neuronal activity/functions as well as with transcriptional activator activity and chromatin binding comparing DS and isogenic cells (Supplementary Figure S6). Interestingly, disease ontology analysis of PAX6 binding revealed several terms associated with disorders of interest: developmental disorders of mental health, including autism spectrum disorder, epilepsy syndrome, and mood disorders, conditions known to affect individuals with DS (Figure 7D). The changes in PAX6 binding in DS cells provide additional insights into the widespread dysregulation of gene expression that may contribute to deficits in neurogenesis and other facets of neuronal differentiation and function.

## Discussion

Intellectual disability in individuals with DS is multifactorial, beginning with significant changes in brain development as early as the second trimester. Reduced neurons, increased astroglia, and delayed cortical lamination indicate impaired neurogenesis in DS brain development. In our study, we integrated mouse and human iPSCs to develop a robust *in vitro* DS neurogenesis model and found that impaired neurogenesis in DS is due to a biphasic cell cycle defect.

Mouse models of DS, segmentally trisomic for mouse genes homologous to those on human chromosome 21, have been examined to understand early changes in brain development. This approach allows it to explore brain development changes *in vivo* and link changes to the responsible gene and mechanisms. Among these mouse models of DS, Ts65Dn is the most studied mouse model. The Ts65Dn mouse model shows microcephaly and reduced neurogenesis during prenatal brain development. Interestingly, it was found that during Ts65Dn brain development, there is a growth delay in cortical walls. In addition, this study also found an increased number of Tbr2+ neural progenitor cells in Ts65Dn SVZ compared to euploid mice (Chakrabarti et al., 2007). These observations indicated increased proliferation of neural progenitor cells in a late phase in the Ts65Dn mouse compared to its euploid counterpart.

Recently, human iPSCs-based modeling has provided an alternative model for exploring fetal DS brain development. Indeed, it has been shown that neural differentiation of hiPSCs displays stages resembling those in the fetal brain at age ∼9–20 gestational week (GW) (Mariani et al., 2012; Espuny-Camacho et al., 2013; Nicholas et al., 2013; Brennand et al., 2015)—i.e., at a stage in which changes in neurogenesis are suggested to characterize DS. In early studies, a few interesting observations have been made, including synaptic deficit (Weick et al., 2013), presence of amyloid plaques (Shi et al., 2012) as well as reduced neurogenesis (Hibaoui et al., 2014). Among the studies showing impaired DS neurogenesis, reduced proliferation of neural progenitors has been reported to cause impaired neurogenesis. However, reduced proliferation alone could not explain increased astroglia production (Guidi et al., 2008) or recovery of the number of neurons observed in the DS visual cortex post-birth (Wisniewski et al., 1984).

To address the etiology of disordered brain development in DS, we noted the advantages of integrating studies in mouse and human cells and carried out studies using iPSCs from the Ts65Dn mouse (Ts65Dn miPSCs) as well as humans (DS hiPSCs). Using this approach, we established robust stem cell models of DS and elucidated cellular and molecular events correlated with reduced neurogenesis.

We used previously published neural differentiation protocols, which rely on the intrinsic properties of pluripotent stem cells and are similar for mouse and human iPSCs (Gaspard et al., 2008; Espuny-Camacho et al., 2013). We found reduced neural differentiation of mouse and human DS stem cells; findings were consistent with mouse and human brain studies (Contestabile et al., 2007; Larsen et al., 2008) and *in vitro* studies using brain-derived human neural progenitors (Bhattacharyya et al., 2009). In addition, our findings show delayed differentiation of DS PAX6+ cells consistent with earlier reports (Jiang et al., 2013). In the mouse iPSCs-based DS neurogenesis model, we analyzed the effect of delayed cell cycle exit using Ki67 and DCX staining. DCX is expressed in migrating and differentiating neurons (Francis et al., 1999) and its mutation is associated with the X-linked lissencephaly (Gleeson et al., 1998). Its reduced expression in the Ts65Dn cells, along with expression of Ki67, showed delayed neural differentiation of the Ts65Dn cells. On the other hand, the human iPSCs-based model was analyzed using PAX6+ cells. PAX6 is a marker for neural progenitor cells and a determinant of human neuroectodermal fate (Zhang et al., 2010). PAX6 exert high level of control of cortical development, and its mutation or deletion from developing brain caused major brain effects and neurodevelopmental disorders (Manuel et al., 2015). Our analysis found that the generation of PAX6+ cells was delayed in DS. PAX6 analysis combined with Ki67 and BrdU pulse labeling experiment found that in the initial phase, neural progenitor cells show reduced proliferation, while in the late phase, neural progenitor cells show increased proliferation compared to isogenic euploid cells. While in mouse studies, DCX analysis indicated reduced differentiation of neurally committed Ts65Dn cells, PAX6 analysis in human cells indicated that neural progenitor generation is also delayed in DS cells. Furthermore, consistent with studies performed using human DS brain-derived neural progenitors (Cairney et al., 2009), we found dysregulation of Notch and Wnt pathways in hiPSCs-derived DS progenitors. Indeed, a recent study published during this work also found upregulated Notch pathways in human iPSCs based DS neurogenesis model (Czerminski and Lawrence, 2020). These results demonstrate that utilizing intrinsic properties of pluripotent stem cells enables studies to confirm and expand upon previous studies in both human and mouse tissues.

Remarkably, DS progenitors displayed two defects in cell cycle regulation during neural differentiation salient for defective neurogenesis. In an early deviation from isogenic euploid, an increase in DS cells in G0 was linked with reduced proliferation of DS neural progenitor cells. A second deviation was apparent concerning entry and exit from the S-phase. Increased proliferation of DS neural progenitor has also been observed in a recent hiPSCs-based study (Czerminski and Lawrence, 2020). Both deviations could be responsible for decreased neurogenic potential of DS cells. In the first case, early cell divisions that support the creation of a larger precursor pool may be compromised. In the second, delayed entry into the S-phase, followed by a delayed exit, may result in the delayed exit of progenitors from the cell cycle, resulting in fewer postmitotic neurons. It is noteworthy that studies in human fetal tissue have shown reduced proliferation of neural progenitors (Contestabile et al., 2007), a finding consistent with delayed entry into the S-phase noted herein. Finally, studies in the developing Ts65Dn and Ts16 mouse brains have shown that neural progenitors are retained for extended periods in the S-phase (Haydar et al., 1996; Chakrabarti et al., 2007). Our studies thus point to the sharing of essential phenotypes, including impaired neurogenesis and cell cycle dysregulation, between mouse and human iPSCs and their *in vivo* counterparts in the brain.

Gene expression analysis of late-stage DS progenitors showed that widespread dysregulation of gene expression accompanied the changes in the cell cycle and neurogenesis and was plausibly linked to the changes detected. There was upregulation of *CDK2, CDK6, CCND2, CDCA3,* and *E2F8*. *CDK2* and *CDK6* promote the entry and accumulation of cells in the S phase (Chevalier et al., 1995; Grossel et al., 1999; Alexandrow and Hamlin, 2005; Deng et al., 2010; Koledova et al., 2010; Uchida et al., 2012; Bertoli et al., 2013). In contrast, genes such as *RB1*, *GEMININ,* and *CDT1* were downregulated. *RB1*, a tumor suppressor gene, acts by preventing progression from G1 to the S phase (Dyson, 2016). GEMININ is an inhibitor of DNA replication; early mouse cortical progenitors lacking GEMININ exhibit a longer S phase and a reduced ability to generate early-born neurons (Spella et al., 2011). CDT1 accumulates in G1 and is destabilized after initiating the S-phase to allow for S-phase progression (Nishitani et al., 2001). These results are consistent with an S phase promoting gene expression in DS cells.

Failure to exit the cell cycle by DS progenitors (i.e., maintaining a proliferative state exhibited by more cells in the S-phase) could be responsible for fate alteration of progenitor cells leading to reduced neurons and an increase in astroglia cells. During neural differentiation, radial glial cells are generated from neuroepithelium and function as primary progenitors or neural stem cells (unrestricted NPCs). These cells initially express astroglia markers such as GLAST, BLBP, and GFAP. Most, if not all, neurons are generated from these cells (Kriegstein and Alvarez-Buylla, 2009). Neural and glial cells are generated in a programmed sequence where neural generation precedes glial cells (Qian et al., 2000). A cell cycle hypothesis (Gotz and Huttner, 2005) proposes that time is a crucial factor, where actions of extrinsic or intrinsic factors depend on their duration of action. Thus, unrestricted progenitors need to exit the cell cycle in a timely fashion to commit toward neural lineage. Apical and basal progenitors committed to neural production exhibit a substantially shorter S phase (Arai et al., 2011). Thus, one may speculate that DS progenitors cannot initiate neural differentiation due to their failure to exit the cell cycle quickly with increased adoption of the glial phenotype. Consistent with this view, DS cells exhibited upregulation of Notch and Wnt pathways. Notch and Wnt have been shown to maintain stem cell self-renewal and fate restriction (Iso et al., 2003; Chiba, 2006; Kalani et al., 2008; Nusse, 2008; Cairney et al., 2009; Katreddi et al., 2022). We found that in addition to DLL1, a Notch ligand, and HEY1/2, a target of notch signaling, genes such as SOX9 and NFIA were upregulated. SOX9 regulates Notch downstream targets and associates with NFIA. NFIA has been shown to induce chromatin remodeling by inducing the release of DNMT1 from the GFAP promoter, thereby allowing astrocytic gene expression (Kang et al., 2012). Additionally, interferon pathways may promote astrogenesis by induction of the JAK-STAT pathway (Bonni et al., 1997; Gough et al., 2008). *REST*, which inhibits neuronal gene expression (Huang et al., 1999), was also upregulated in DS cells. *ACTL6B* (*BAF53b*) was one of several downregulated BAF complex genes. *ACTL6B* (*BAF53b*) is part of the neuron-specific BAF chromatin-remodeling complex (Lessard et al., 2007) and plays a role in dendritic outgrowth (Wu et al., 2007), synaptic plasticity and memory (Vogel-Ciernia et al., 2013). Consistently, we found downregulation of neurogenic genes *NFIB* and *POU3F4,* which are activated by PAX6-BAF interaction. Additionally, downregulated oxidative phosphorylation pathways suggest mitochondrial dysfunction that has been proposed to cause reduced neurogenesis while increasing glia production (Diaz-Castro et al., 2015). ChIPseq analysis revealed aberrant PAX6 binding with reduced promoter occupancy in DS cells. This analysis found that while promoter occupancy was reduced (4% vs. 7%), overall binding of PAX6 was increased in DS cells (11620 vs. 3,368). In addition, a previous study has shown a permissive chromatin state in trisomic cells using a DNase hypersensitivity assay (Letourneau et al., 2014). Together, these results indicate that DS cells have defective chromatin packaging. Furthermore, gene ontology analysis of PAX6 binding found an alteration in ionotropic glutamate receptor activity, transcriptional activator activity, chromatin binding, and potassium channel activity that have been shown to affect neurogenesis (Luk et al., 2003; Schlett, 2006; Song et al., 2013).

Impaired neurogenesis due to cell cycle and genomic abnormalities observed in DS cells could be either due to aneuploidy, as proposed (Beach et al., 2017) or due to overexpression of either individual HSA21 genes or their combinatorial effect. For instance, overexpression of DYRK1a may cause the accumulation of DS cells in the G0/G1 phase leading to reduced proliferation (Hammerle et al., 2011). In contrast, overexpression of DYRK1a in combination with DSCR1 has been shown to cause S phase lengthening (Kurabayashi and Sanada, 2013). In addition to DYRK1a and DSCR1, OLIG1/2 (Chakrabarti et al., 2010) and USP16 (Adorno et al., 2013) have been shown to have a role in cell cycle and neurogenesis in DS. Furthermore, HMGN1, expressed on HSA21 and has recently been shown to have a role in B cell lymphoblastic leukemia (Lane et al., 2014), might disrupt chromatin in DS progenitor cells, leading to abnormal PAX6 binding. The altered binding of PAX6 may also lead to cell cycle abnormalities (Sansom et al., 2009). The human cell model employed herein may help to systematically explore a role for aneuploidy using recently developed DS hiPSCs with Robertsonian translocation (Nehra et al., 2022) and the stage-specific molecular mechanisms of increased dose of HSA21 genes in impaired neurogenesis. For instance, Hibaoui et al. found that EGCG treatment, a DYRK1A kinase inhibitor, before starting neural induction of hiPSCs, rescued DS neural differentiation (Hibaoui et al., 2014). However, when applied during neuronal differentiation, EGCG treatment failed to rescue DS neurogenesis. This observation indicated that molecular mechanisms causing DS impaired neurogenesis might differ in the late phase compared to the early phase of neurogenesis. Indeed, our study found two phases; reduced proliferative phase followed by increased proliferative phase. Hibaoui et al. found reduced REST, Notch, and Wnt, consistent with reduced proliferation observed in their study (Hibaoui et al., 2014). On the contrary, our analysis in the late phase, i.e., during the increased proliferative phase, found upregulation of REST, Notch, and Wnt. These differences in stage-specific mechanisms might be relevant for developing effective treatments for DS ID.

In summary, by taking advantage of both systems, i.e., in-depth studies carried out in the Ts65Dn mouse model and DS human iPSCs, we developed a robust *in vitro* model of DS impaired neurogenesis. We have identified that impaired neurogenesis in DS is due to a biphasic cell cycle defect during the neurogenic phase where DS cells, compared to control cells, first go through reduced proliferation followed by increased proliferation. We identified changes in the gene expression and PAX6 binding, which may explain cell cycle defect at the late phase of the neurogenic stage and impaired neurogenesis. Using the human DS/mouse DS model platform will enable future studies to discover shared genes and mechanisms for defective neurogenesis in DS.

## Data Availability

The RNAseq and ChIPseq data discussed have been deposited in NCBI’s Gene Expression Omnibus with accession number GSE95553.
