# Supplementary material for: Biphasic cell cycle defect causes impaired neurogenesis in down syndrome
Source: Front Genet. 2022 Oct 12;13:1007519. doi: 10.3389/fgene.2022.1007519 (PMC9596798; doi:10.3389/fgene.2022.1007519)
Supplement: Supplementary file 1 [file DataSheet2.PDF]

Table S2: Antibodies used in the study

Name	Source	Catalogue #
**Anti-Doublecortin antibody (DCX)**	Abcam	Ab18723
**Anti-Glial Fibrillary Acidic Protein (GFAP)**	Dako Cytomation	Z 0334
**Anti-Neuronal Class III b-Tubulin (TUBB3)**	Covance (Biolegend)	MMS-435P
**Anti-Fox3/NeuN**	Covance (Biolegend)	MCS-1B7
**Anti-Ki67**	BD Pharmingen	550609
**Anti-Map2A,2B**	Millipore	MAB378
**Anti-PAX6**	Covance (Biolegend)	PRB-278P
**Rabbit Polyclonal Isotype**	Covance (Biolegend)	CTL-4112
**Anti-AFP (C-19)**	Santa Cruz	sc-8108
**Anti Alpha Actin (Smooth Muscle)**	Millipore	MABT 381
**Anti-ALDH1L1**	Neuromics	RA22119
**Anti-Pax6 (ChIP)**	Millipore	AB2237
**BrdU Flow Kits**	BD Pharmingen	559619
**Fluorescent mouse ES/iPS cell Kit**	Millipore	SCR077
**Fluorescent Human ES/iPS Cell Kit**	Millipore	SCR078
