# Supplementary figures and images for: Biphasic cell cycle defect causes impaired neurogenesis in down syndrome
Source: Front Genet. 2022 Oct 12;13:1007519. doi: 10.3389/fgene.2022.1007519 (PMC9596798; doi:10.3389/fgene.2022.1007519)

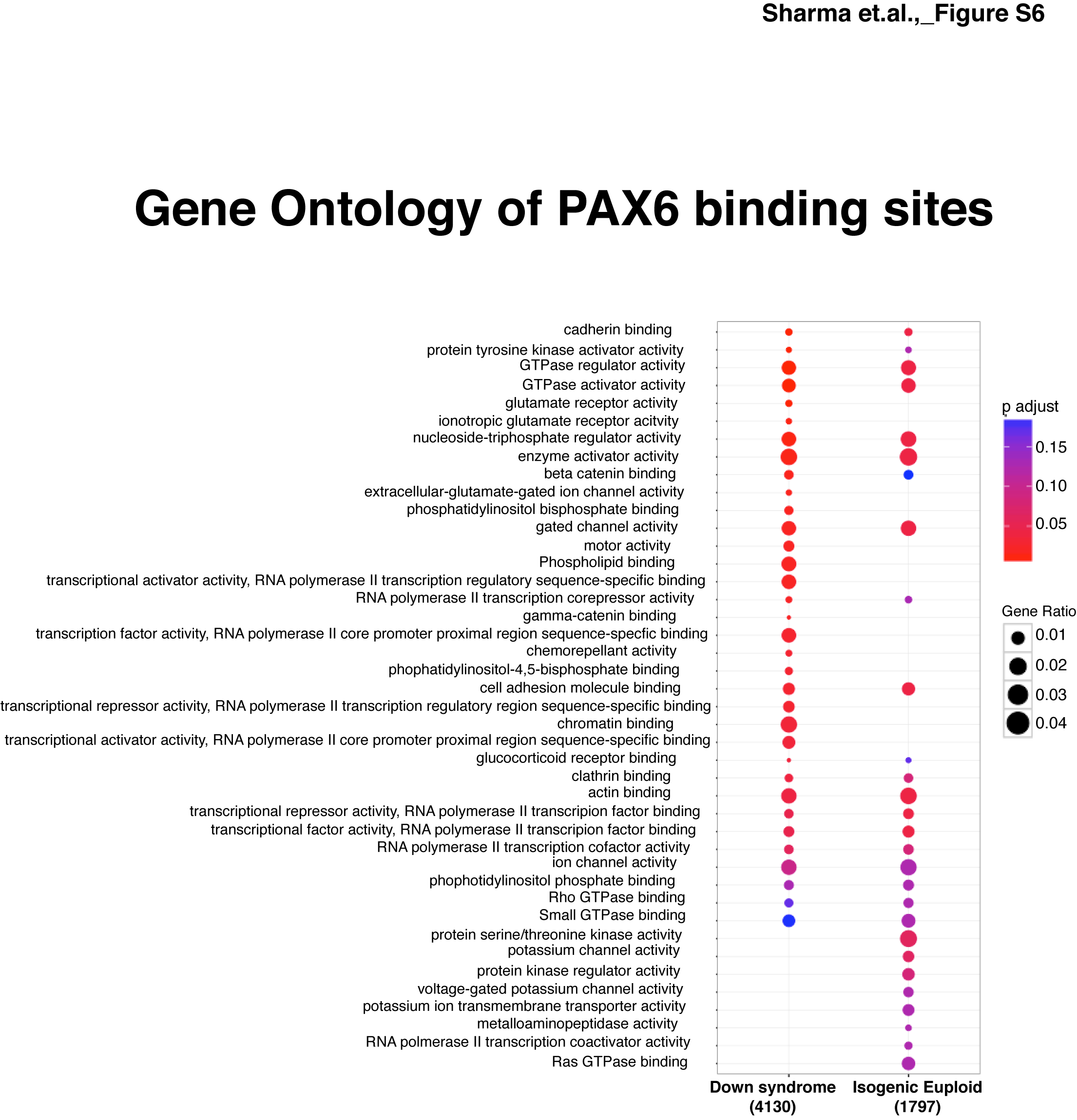

Supplement: Supplementary file 2 [file Image6.TIF]

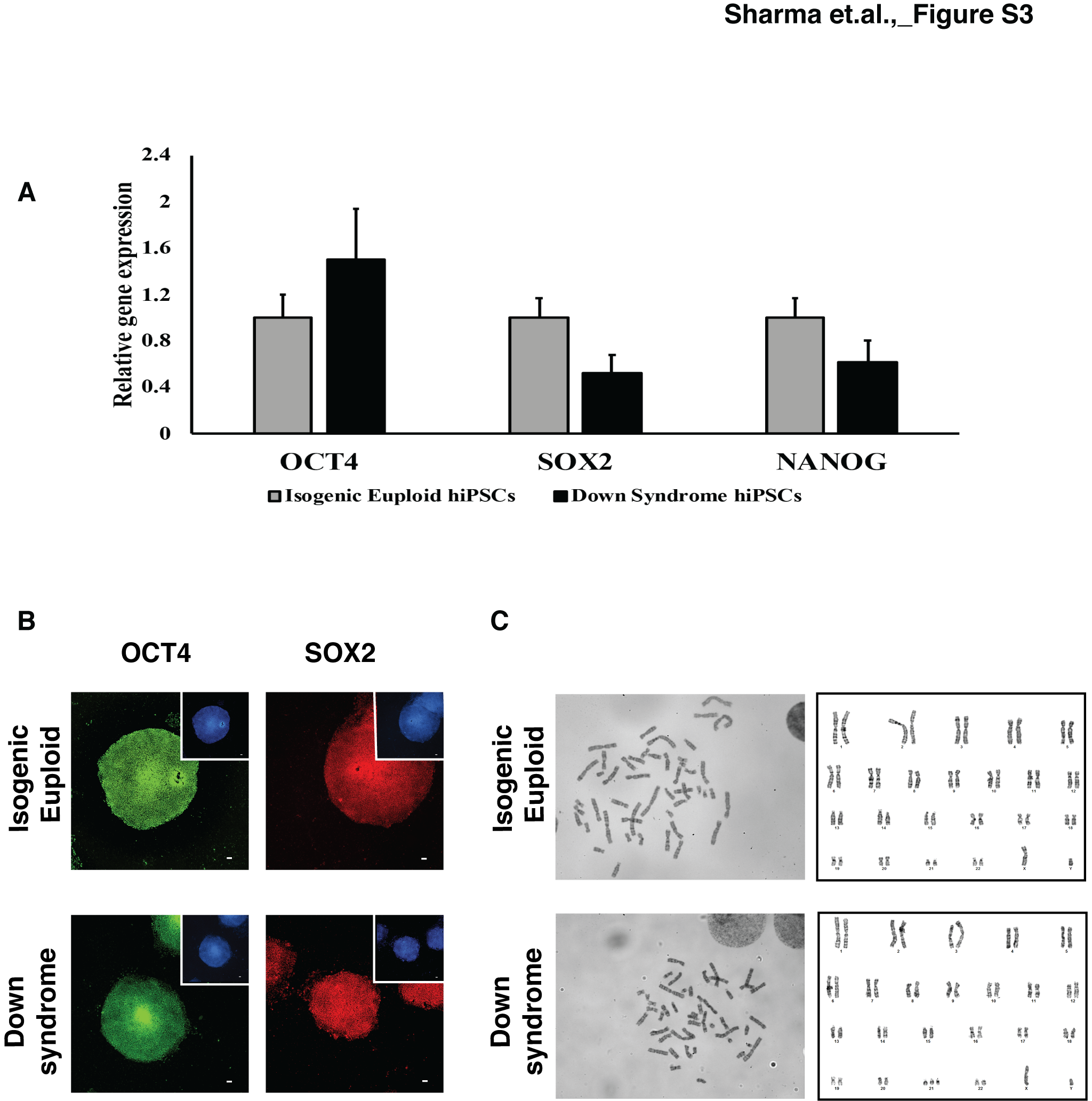

Supplement: Supplementary file 3 [file Image3.TIF]

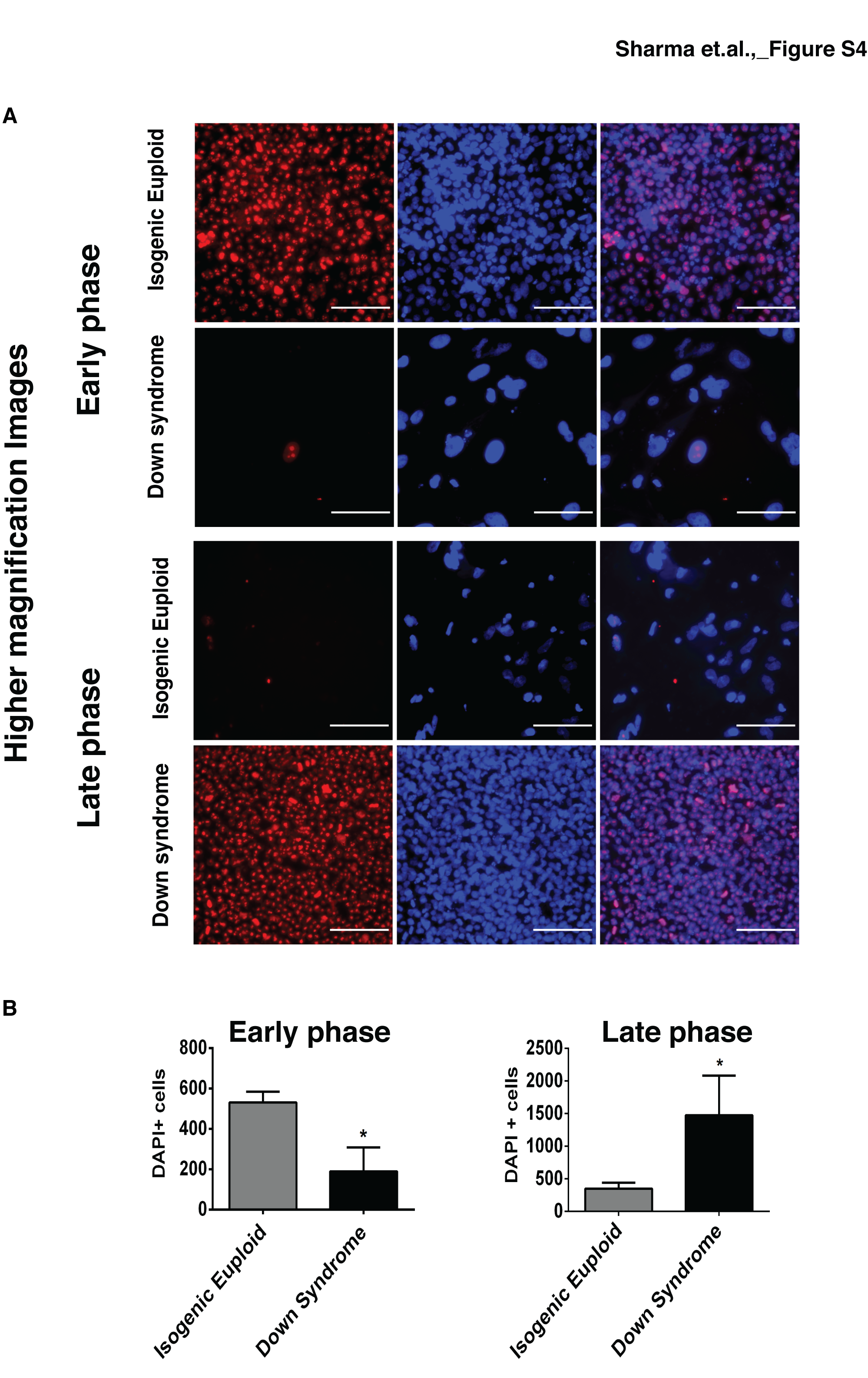

Supplement: Supplementary file 4 [file Image4.TIF]

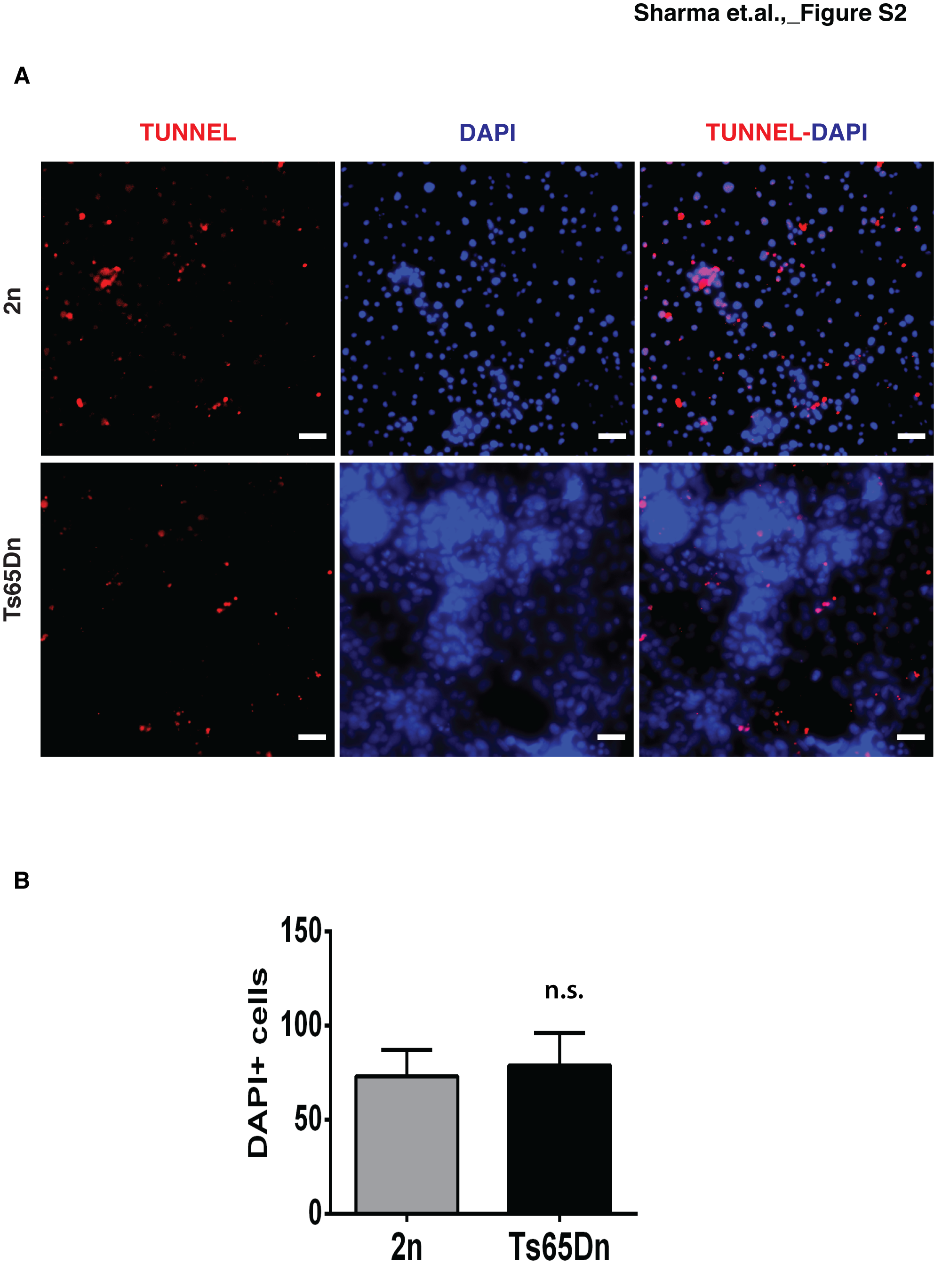

Supplement: Supplementary file 5 [file Image2.TIF]

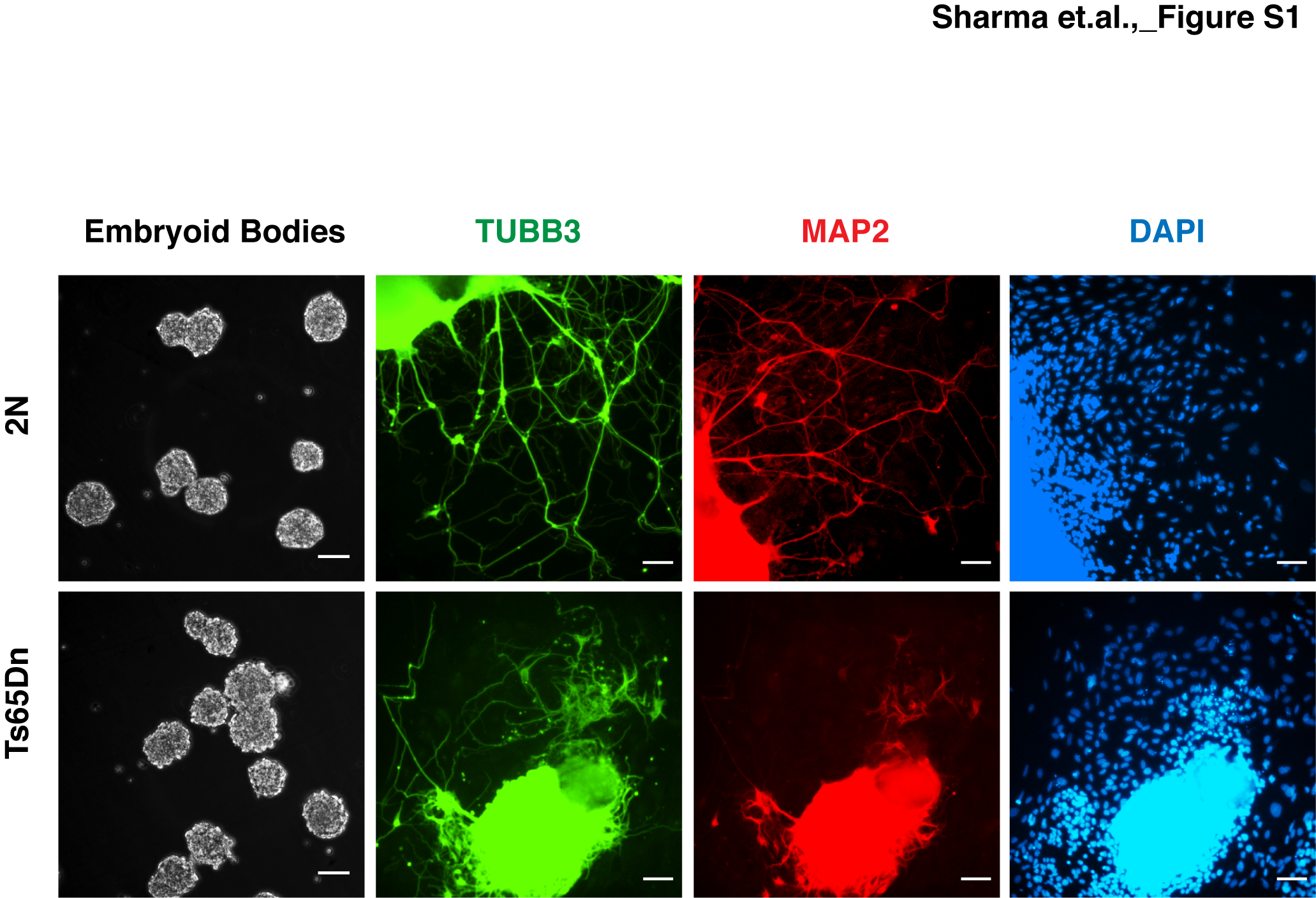

Supplement: Supplementary file 6 [file Image1.TIF]

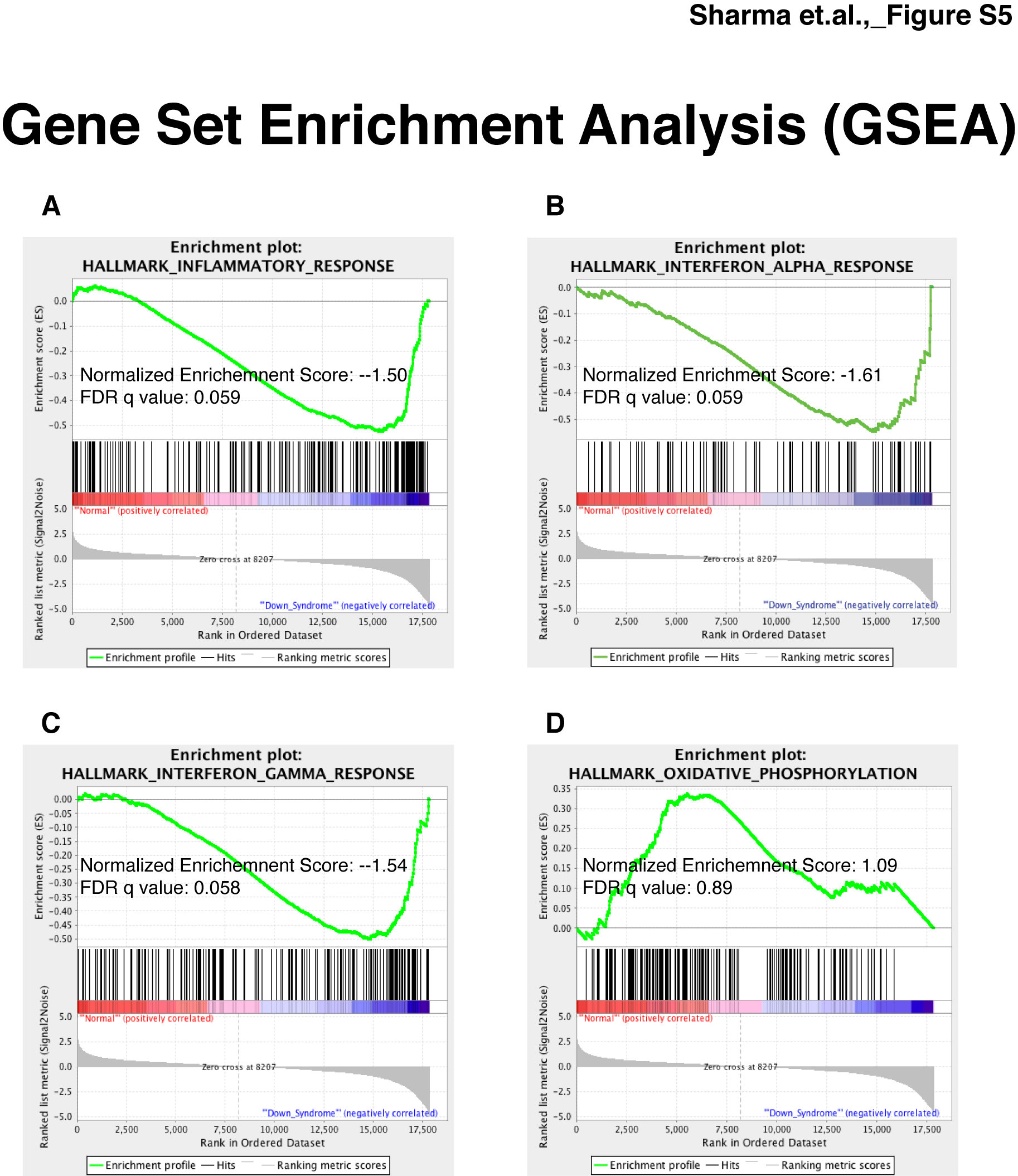

Supplement: Supplementary file 9 [file Image5.TIF]
