# Supplementary material for: Biphasic cell cycle defect causes impaired neurogenesis in down syndrome
Source: Front Genet. 2022 Oct 12;13:1007519. doi: 10.3389/fgene.2022.1007519 (PMC9596798; doi:10.3389/fgene.2022.1007519)
Supplement: Supplementary file 7 [file DataSheet3.PDF]

Figure S1. Analysis of embryoid mediated (EBs) neural differentiation of 2N and Ts65Dn miPSCs. 2N and Ts65Dn iPSCs were cultured in suspension for three days to generate EBs. EBs mediated neurons were analyzed by TUBB3 (green) and MAP2 (red) expression with corresponding DAPI staining. The scale bar is 50µM. (Linked to Figure 2).

Figure S2. Tunnel Assay analysis of 2N and Ts65Dn NPCs at DIV12 of neurodifferentiation. (A) Representative images of TUNNEL and corresponding DAPI images and their overlay. (B) Quantification of DAPI+ cells at DIV 26 for analysis shown in figure 3 for Ki67 and DCX expression. Data are represented as mean ± SEM where N=3. A total of 3 independent experiments were performed using the same batch of iPSCs. *p<0.05, **p<0.01, ***p<0.001, n.s. non-significant. The scale bar is 50µM. (Linked to Figure 3)

Figure S3. Isogenic pair of Euploid and Down Syndrome hiPSCs characterization. (A) QRT-PCR analysis of Isogenic Euploid and Down Syndrome hiPSCs for relative gene expression of pluripotency markers OCT4, SOX2, and NANOG. (B) ICC to check the expression of pluripotency-associated markers OCT4 and SOX2. Scale bar is 100µM. (C) Karyotype analysis of Isogenic Euploid and Down Syndrome hiPSCs and their raw images. 60X magnification. (Linked to Figure 4)

Figure S4. Analysis of human neural progenitor cells during neural differentiation

(A) Higher magnification representative images of Ki67 along with corresponding DAPI images and their overlay images at the early phase (DIV 18) and late phase (DIV 28) of stage 1 of neurodifferentiation. (B) Quantification of DAPI+ cells at the early and late phase of stage 1 of neurodifferentiation. Data are represented as mean ± SEM where N=3. A total of 3 independent experiments were performed using the same batch of iPSCs. *$p<0.05$, **$p<0.01$, ***$p<0.001$, n.s. non-significant. The scale bar is 100μM. (Linked to Figure 5)

Figure S5. RNAseq analysis of global gene expression of Down syndrome and isogenic euploid DIV 24 progenitor cells. Enrichment of Inflammatory (A) Interferon alpha (B), Interferon Gamma (C), and Oxidative phosphorylation (D) pathways using hallmark gene set analysis in GSEA. FDR, false discovery rate. (Linked to Figure 6)

Figure S6. Gene ontology analysis using PAX6 binding sites. (linked to Figure 7).
