# Supplementary material for: Biphasic cell cycle defect causes impaired neurogenesis in down syndrome
Source: Front Genet. 2022 Oct 12;13:1007519. doi: 10.3389/fgene.2022.1007519 (PMC9596798; doi:10.3389/fgene.2022.1007519)
Supplement: Supplementary file 8 [file DataSheet1.PDF]

Table S1: Selected regulators of Cell cycle and fate determinants

Genes	Log2FC	P-adj	Function
SOX9	0.8	2.4E-12	Gliogenesis
NFIA	1.53	8.2E-19	Gliogenesis
REST	0.63	1.73E-37	Neural suppression
CDCA3	0.31	1.42E-07	S-phase accumulation
E2F8	0.20	0.02	S phase accumulation
GEMININ	-0.32	2.52E-11	Inhibitor of DNA replication
CDT1	-0.33	0.01	Prevent re-replication of DNA
RB1	-0.21	0.002	Negative regulator of cell cycle
